# Investigation of neglected protists *Blastocystis* sp. and *Dientamoeba fragilis* in immunocompetent and immunodeficient diarrheal patients using both conventional and molecular methods

**DOI:** 10.1371/journal.pntd.0009779

**Published:** 2021-10-06

**Authors:** Fakhriddin Sarzhanov, Funda Dogruman-Al, Monica Santin, Jenny G. Maloney, Ayse Semra Gureser, Djursun Karasartova, Aysegul Taylan-Ozkan

**Affiliations:** 1 Division of Medical Parasitology, Department of Medical Microbiology, School of Medicine, Gazi University, Ankara, Turkey; 2 Akhmet Yassawi International Kazakh-Turkish University, Faculty of Medicine, Turkestan, Kazakhstan; 3 Environmental Microbial and Food Safety Laboratory, Agricultural Research Service, United States Department of Agriculture, Beltsville, Maryland, United States of America; 4 Department of Medical Microbiology, School of Medicine, Hitit University, Corum, Turkey; 5 Department of Medical Microbiology, Faculty of Medicine, TOBB- University of Economics and Technology, Ankara, Turkey; University of Pittsburgh, UNITED STATES

## Abstract

**Introduction:**

The clinical significance of *Blastocystis* sp. and *Dientamoeba fragilis* in patients with gastrointestinal symptoms is a controversial issue. Since the pathogenicity of these protists has not been fully elucidated, testing for these organisms is not routinely pursued by most laboratories and clinicians. Thus, the prevalence of these organisms and the subtypes of *Blastocystis* sp. in human patients in Turkey are not well characterized. This study aimed to determine the prevalence of *Blastocystis* sp. and *D*. *fragilis* in the diarrheic stool samples of immunodeficient and immunocompetent patients using conventional and molecular methods and to identify *Blastocystis* sp. subtypes using next generation sequencing.

**Material and methods:**

Individual stool specimens were collected from 245 immunodeficient and 193 immunocompetent diarrheic patients between March 2017 and December 2019 at the Gazi University Training and Research Hospital in Ankara, Turkey. Samples were screened for *Blastocystis* sp. and *D*. *fragilis* by conventional and molecular methods. Molecular detection of both protists was achieved by separate qPCRs targeting a partial fragment of the *SSU* rRNA gene. Next generation sequencing was used to identify *Blastocystis* sp. subtypes.

**Results:**

The prevalence of *Blastocystis* sp. and *D*. *fragilis* was 16.7% and 11.9%, respectively as measured by qPCR. The prevalence of *Blastocystis* sp. and *D*. *fragilis* was lower in immunodeficient patients (12.7% and 10.6%, respectively) compared to immunocompetent patients (21.8% and 13.5%, respectively). Five *Blastocystis* sp. subtypes were identified and the following subtype distribution was observed: ST3 54.4% (n = 37), ST2 16.2% (n = 11), ST1 4.4% (n = 3), ST6 2.9% (n = 2), ST4 1.5% (n = 1), ST2/ST3 11.8% (n = 8) and ST1/ST3 8.8% (n = 6). There was no statistically significant difference in the distribution of *Blastocystis* sp. subtypes between immunocompetent and immunodeficient patients.

**Conclusion and recommendation:**

Our findings demonstrated that *Blastocystis* sp. and *D*. *fragilis* are commonly present in immunocompetent and immunodeficient patients with diarrhea. This study is the first to use next generation sequencing to address the presence of *Blastocystis* sp. mixed subtypes and intra-subtype variability in clinical samples in Turkey.

## Author summary

*Blastocystis* sp. and *Dientamoeba fragilis* are single-cell parasites of the human intestine which are common worldwide and reported in cases with gastrointestinal symptoms. However, the role of *Blastocystis* sp. and *D*. *fragilis* in patients with gastrointestinal symptoms is still controversial because their presence is not always associated with symptoms. As some intestinal parasitic infections can cause severe illness in immunocompromised individuals careful consideration of intestinal protist infection is warranted. However, testing for *Blastocystis* sp. and *D*. *fragilis* is not routinely carried out by most laboratories and clinicians. Therefore, this study aimed to determine the prevalence of *Blastocystis* sp. and *D*. *fragilis* in immunocompetent and immunosuppressed patients with diarrhea by conventional and molecular methods. Both *Blastocystis* sp. and *D*. *fragilis* were detected more frequently by quantitative polymerase chain reaction than by conventional methods. Next generation sequencing was used to characterize the diversity and frequency of *Blastocystis* sp. subtypes and mixed subtypes in patients in Turkey. Five *Blastocystis* sp. subtypes (ST1, ST2, ST3, ST4 and ST6) were detected. ST3 was the most frequent subtype in both immunocompetent and immunosuppressed patients. Mono-infections were more common than mixed subtype infections. Our findings showed that *Blastocystis* sp. and *D*. *fragilis* are commonly present in immunocompetent and immunodeficient patients with diarrhea.

## Introduction

Diarrhea is one of the most widespread gastrointestinal symptoms and is a common problem in immunosuppressed patients. The spectrum of pathogens that cause diarrhea in immunosuppressed patients is significantly different from those in patients with a normal immune system. In fact, in immunocompromised individuals, there is a higher risk for opportunistic pathogen infections. Such pathogens include *Cryptosporidium* spp. and *Cystoisospora belli*, which are classical opportunistic parasites commonly found in immunosuppressed diarrheal patients [1–5]. *Blastocystis* sp. and *Dientamoeba fragilis* are cosmopolitan intestinal protists commonly reported in people with and without symptoms [6–9]. Gastrointestinal symptoms, such as diarrhea, abdominal pain, and irritable bowel syndrome, have been associated with their infections/colonizations [10–12]. However, because both protists are also commonly observed in asymptomatic people, their clinical significance is still controversial [8, 12, 13]. The pathogenic potential of *Blastocystis* sp. and *D*. *fragilis* is not clear, but there are reports of their presence in immunocompromised individuals (cancer or HIV-infected patients) associated with gastrointestinal symptoms suggesting that they could be a relevant threat to immunocompromised populations [14, 15].

*Blastocystis* sp. is estimated to colonize more than one billion people worldwide [16]. Prevalence ranges of 0.5% to 100% from developing countries [17–23] and 1.2% to 35.2% from developed countries [24–28] have been reported. Currently, based on analysis of the small subunit (*SSU*) of the ribosomal RNA (rRNA) gene, 28 subtypes (STs) have been proposed in birds and mammals [7, 29–31]. Of those, 24 subtypes (ST1-ST17, ST21, ST23-ST28) are currently acknowledged as valid subtypes [30, 32]. Twelve subtypes (ST1-ST10, ST12, and ST14) have been found in humans with different levels of prevalence [33–38]. It has generally been reported that ST1-ST4 are more commonly seen in humans, whereas ST5-ST10, ST12, and ST14 in humans likely represent the consequence of a zoonotic transmission event. [33, 35–38].

*Dientamoeba fragilis* has been reported in humans with a worldwide distribution [12, 39]. Most studies have been conducted in industrialized countries where prevalence ranged from 0.3% to 82.9% [40–44]. Less is known from the developing world, but prevalence is reported to range from 0% to 60.6% [45–49]. There are two described genotypes of *D*. *fragilis*, named 1 and 2, which were defined using molecular analysis of restriction fragment length polymorphisms in the *SSU* rRNA gene [50]. Potential for zoonotic transmission has been suggested based on the few reports of *D*. *fragilis* in non-human hosts that include non-human primates (gorilla), pigs, and companion animals (dogs and cats). [51–55].

The most common parasitological examination methods used in clinical laboratories to detect *Blastocystis* sp. and *D*. *fragilis* are based on microscopy: direct smear (Native-Lugol examination), formalin-ethyl acetate concentration technique (FECT), and permanent staining. However, these methods are known to be insufficient for the definitive diagnosis of these two protists [56–59]. For *Blastocystis* sp., culture from stool samples are significantly more sensitive than direct microscopic examination for the detection, but stool cultures can be time consuming making them not practical for diagnosis when a quick turnaround is needed [56, 60]. Molecular techniques are progressively replacing microscopy for diagnosis of intestinal parasites, and they are the first-line diagnostic method in laboratories particularly in industrialized countries [61]. Molecular epidemiology studies of *Blastocystis* sp. and *D*. *fragilis* have clearly demonstrated that molecular screening methods are needed in accurately detecting the presence of these protists in stool samples [12, 56, 57, 62, 63]. The use of molecular methods to improve detection of *Blastocystis* sp. and *D*. *fragilis* in stool samples is crucial as it is challenging to identify these parasites by microscopy. By improving detection, we also improve our understanding of their epidemiology.

For *Blastocystis* sp., it is also key to identify subtypes and intra-subtype variability to understand its public health significance and pathogenicity. Several methods have been used to describe *Blastocystis* sp. mixed subtype infections. Next generation sequencing (NGS) provides a powerful tool for *Blastocystis* sp. detection that allows: subtype identification, detection of mixed subtypes within a sample, detection of low-abundance subtypes, and intra-subtype variations [8, 30, 64]. The aim of this study was to detect the prevalence of *Blastocystis* sp. and *D*. *fragilis* in immunocompetent and immunosuppressed patients with diarrhea by conventional and molecular methods. We also use NGS to characterize the prevalence of *Blastocystis* sp. subtypes and mixed subtypes in these patients. This is the first study to use next-generation sequencing technology to investigate *Blastocystis* sp. subtypes in Turkey.

## Material and methods

### Ethics statement

All study procedures, informed consent forms, and epidemiological questionnaires involved in the study were approved by the Ethics Commission of Gazi University (09.05.2017/05). Written informed consent was obtained from the participants.

### Study population and collection of samples

**Fig 1:**
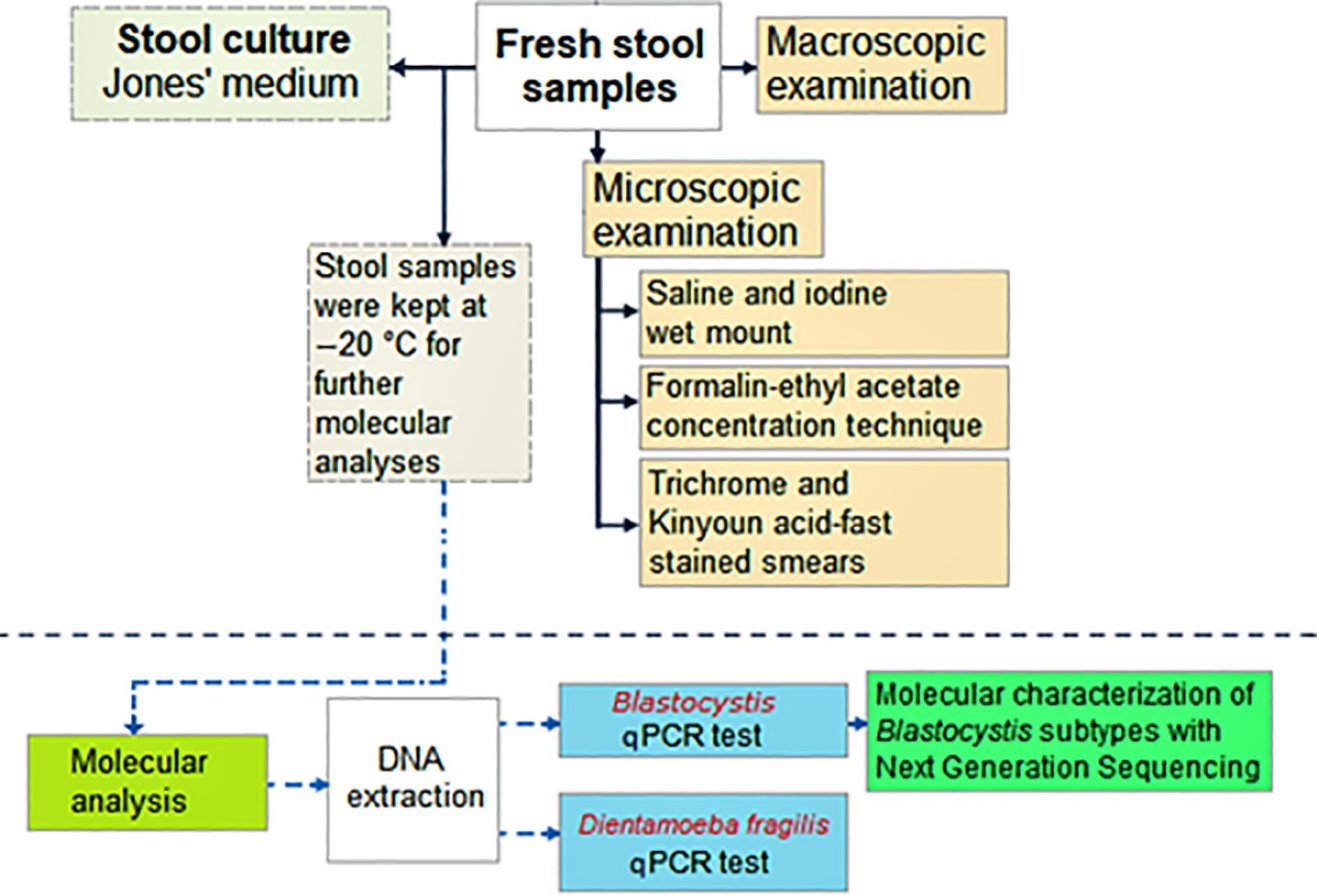
Flow chart used to process stool specimens. Diagram showing the flow of clinical samples, diagnostic, and molecular identification procedures followed in the present study.

Individual stool samples from 438 outpatients were collected between March 2017 and December 2019 at the Gazi University Training and Research Hospital in Ankara, Turkey. The inclusion criteria for the study was to be adult patients (18 years and older) with diarrhea. Diarrhea was defined according to the Bristol Stool Form Scale. Stool samples were examined macroscopically and compared to the Bristol stool chart, and stools consistent with type 6 (fluffy pieces with ragged edges, a mushy stool) and type 7 (watery, no solid pieces, entirely liquid) were considered diarrhea [65]. Patients included in the immunodeficiency patient group were those patients treated at hematology, oncology, rheumatology, nephrology, and bone marrow transplantation units. Patients included in the immunocompetent patient group where those treated at gastroenterology and other clinics with the complaint of diarrhea and with no known immunodeficiency. Exclusions to participate in the study included patients who have inflammatory bowel disease, irritable bowel syndrome, urticaria, under antibiotic treatment, or those that had a colonoscopy in the last three months. Additionally, any patients found positive to Adenovirus and Rotavirus (RIDA QUICK Rotavirus/Adenovirus Combi test, R-biopharm, Germany) or Salmonella/Shigella (culture) were also excluded from the study. Of samples which met the criteria for inclusion in this study, 245 (56.3%) were immunodeficient diarrheal patients and 193 (43.7%) were immunocompetent diarrheal patients. Among immunodeficient patients, four were from rheumatology (immunosupresive treatment recipients), nine were from nephrology, 56 received bone marrow transplant, 70 were from medical oncology, and 106 were from hematology clinics (S1 Table). One hundred thirty-six (55.52%) patients with immunodeficiency were female and 109 (44.48%) were male. The age of the immunodeficient patients ranged from 18 to 85 years with a median age of 55 years. Immunocompetent patients consisted of 99 (51.30%) females and 94 (48.70%) males. The ages of the immunocompetent patients ranged from 1 to 84 years, and the median age was 41 years (p<0,001). All collected stool samples were independently screened for the presence of enteric parasites by conventional (Native-Lugol examination, FECT, trichrome, and acid-fast staining) and molecular methods for the detection of *Blastocystis* sp. and *D*. *fragilis*. A flow chart of parasite detection methods used in this study is depicted in Fig 1. *Blastocystis* sp. and *D*. *fragilis* positive stool samples were screened by ELISA test for *E*. *histolytica*, *G*. *duodenalis* and *Cryptosporidium* spp. according to manufacturer’s recommendations (E. HISTOLYTICA II, TECHLAB, USA; GIARDIA II, TECHLAB, USA; CRYPTOSPORIDIUM II, TECHLAB, USA, respectively).

### Microscopy

Fresh stool samples were immediately examined microscopically by preparing saline and iodine wet mounts to test for the presence of enteric parasites. Saline and iodine wet mounts were prepared by mixing a small volume of stool with a drop of physiological saline or Lugol’s iodine (diluted 1:5 with distilled water) on a glass microscope slide and placing a coverslip over the mixture [66]. Entire coverslips were examined systematically at 10X and 40X magnification under a light microscope (CX31, Olympus, Japan).

The formalin-ethyl acetate concentration technique was conducted for all stool samples as previously described [67]. Briefly, for each sample, 3 ml of ethyl-acetate solution were added to 10 ml of filtered stool suspension and the tubes were vigorously shaken and centrifuged at 500 × g for 10 minutes. After centrifugation, the supernatant was discarded and the pellet was placed on a microscope slide, covered with a coverslip, and examined microscopically as described above. Additionally, trichrome and Kinyoun’s acid-fast stained smears were prepared from all stool samples, after parasite concentration, and examined using a 100X immersion objective by screening a minimum of 200–300 fields [66].

### *Blastocystis* sp. culture

Fresh stool specimens were cultured in 2 ml Eppendorf tubes containing Jones’ medium with 10% horse serum and incubated at 37°C for 48–72 hours then examined microscopically to detect *Blastocystis* sp. [60].

### DNA extraction

Genomic DNA was isolated from all stool samples. First, approximately 200 mg of each fecal sample was lysed using a tissue homogenizer (Qiagen TissueLyser LT, Hilden, Germany) for 15 minutes by adding 200 mg of acid-washed glass beads prior to DNA extraction then processed according to the manufacturer’s recommendation using a QiaAmp DNA Stool mini-kit (Qiagen, Hilden, Germany). DNA was eluted in 100 μl elution buffer provided with the kit. DNA extraction was performed weekly. DNA was stored at -20°C until molecular analyses were performed.

### Molecular detection of *Blastocystis* sp. and D. fragilis

All stool samples were tested by quantitative polymerase chain reaction (qPCR) for both *Blastocystis* sp. and *D*. *fragilis* every two weeks. Molecular detection of *Blastocystis* sp. was achieved by a qPCR method to specifically amplify a 118-bp fragment of the *SSU* rRNA gene of the parasite [68]. Amplification reactions (25 μl) contained 12.5 μl of PCR Master Mix (Thermo Fisher Scientific, Waltham, MA, USA), 0.5 μM of the primer pair Blasto_FWD_F5/Blasto_R_F2, 0.3 μM *of* probe (S2 Table), and 2 μl of template DNA. Cycling parameters were 95°C for 3 min, and 40 cycles of denaturation at 95°C for 15 s followed by annealing and extension at 57°C for 1 min.

Detection of *D*. *fragilis* was achieved by a qPCR protocol amplifying a 78-bp fragment of the *SSU* rRNA gene of the parasite [69]. Reaction mixes (25 μl) consisted of 12.5 μl PCR of Master Mix (Thermo Fisher Scientific), 0.5 μM of the primer pair DF3/DF4, 0.3 μM of probe (S2 Table), and 2 μl of template DNA. Cycling parameters were the same as for *Blastocystis* sp.: 95°C for 3 min, and 40 cycles of denaturation at 95°C for 15 s followed by annealing and extension at 57°C for 1 min. For all *Blastocystis* sp. and *D*. *fragilis* negative qPCR results, these DNAs were diluted tenfold with 1xTE buffer and then qPCR was repeated.

Positive (target DNA previously identified as *Blastocystis* sp. or *D*. *fragilis* by DNA sequencing) and negative (sterile water) controls were included in each run. Amplification reaction was carried out in a Rotor-Gene 6000 real-time cycler (Rotor-Gene Q, Germantown, MD, USA).

### Molecular characterization of *Blastocystis* sp. subtypes

To detect *Blastocystis* sp., a *ca*. 500 base pair fragment of the *Blastocystis* sp. *SSU* rRNA gene, which contains a variable region suitable for subtyping, was amplified by PCR [70]. PCR products were analyzed using a QIAxcel (Qiagen, Valencia, CA, USA). All positive samples were used to conduct next generation amplicon sequencing and libraries were prepared as previously described [64]. Briefly, all positive samples were amplified by PCR using primers ILMN_Blast505_532F and ILMN_Blast998_1017R. These primers amplify a region of the *SSU* rRNA gene and are identical to Blast505_532F/Blast998_1017R [70], except for containing the Illumina overhang adapter sequences on the 5′ end. PCR conditions were as follows: 95°C for 4 min, 35 cycles of 95°C for 30 s, 54°C for 30s, and 72°C for 30 s, and a final elongation step at 72°C for 5 min. Each 25 μl PCR reaction contained 2.5 μl template DNA, 12.5 μl 2X KAPA HiFi HotStart ReadyMix (KAPABioSystems, Cape Town, South Africa), 2.5 μl BSA (0.1 g/10 ml),1 μM of each primer, 7.75 μl H_2_O, and 1.25 ul of BSA (0.1 g/10 ml). Final libraries were quantified using the Quant-iT dsDNA Broad-Range Assay Kit (ThermoFisher, Waltham, MA, USA) on a SpectraMax iD5 (Molecular Devices, San Jose, CA, USA) prior to normalization. A final pooled library concentration of 8 pM with 20% PhiX control was sequenced using Illumina MiSeq 600 cycle v3 chemistry (Illumina, San Diego, CA, USA). Paired-end reads were processed and analyzed with an in-house pipeline that uses the BBTools package v38.22 [71], VSEARCH v2.8.0 [72], and BLAST+ 2.7.1. After removing singletons, clustering, and the assignment of centroid sequences to operational taxonomic units (OTU) was performed within each sample at a 98% identity threshold. Only those OTUs with a minimum of 100 sequences were retained. All OTUs were assigned a *Blastocystis* sp. subtype based on the best match by BLAST search in the GenBank database. The nucleotide sequences for unique sequences obtained in this study have been deposited in GenBank under the accession numbers MW728054- MW728093.

### Data analysis

Between-group differences in baseline characteristics and parasite prevalence were calculated using Pearson chi-square test, Fisher’s exact test, and Kruskal-Wallis rank test in STATISTICA 12.0 (StatSoft, Tulsa, OK, USA). Proportion confidence limits were carried out using software available at http://openepi.com/Proportion/Proportion.htm. Cohen’s kappa index for intertest agreement was calculated using software available at (http://openepi.com/DiagnosticTest/DiagnosticTest.htm). Kappa considered values < 0 as indicative of no agreement,0–0.20 as slight agreement, 0.21–0.40 as fair agreement, 0.41–0.60 as moderate agreement, 0.61–0.80 as substantial agreement, and 0.81–1 as almost perfect agreement [73]. Medians and means of cycle threshold (Ct) values were calculated and a two-tailed Student t test for comparison of medians was carried out using STATISTICA version 12.0. Odds ratios (OR) and their 95% confidence intervals (CI) were calculated by univariate and multivariable analyses using logistic regression models to assess the association between potential risk factors. Exact logistic regression was used to calculate univariate odds ratios to avoid division by zero (https://stats.blue/Stats_Suite/logistic_regression_calculator.html). The qPCR was considered the reference test to compare results obtained by conventional methods for detection of *Blastocystis* sp. and *D*. *fragilis*. The statistical significance level was considered p <0.05 for all analyses.

## Results

A total of 438 diarrheal patients, including immunocompromised (n = 245) and immunocompetent (n = 193) were recruited to participate in this study. There was no statistically significance between the two groups according to sex. However, there was a difference between two groups according to age. The median age was higher in patients with immunodeficiency than in immunocompetent patients. This observation could be due to the emergence of immunodeficiency as age progresses. Microscopic examination of the samples allowed for identification of other parasitic or commensal protozoans present in the samples in addition to *Blastocystis* sp. and *D*. *fragilis*. Using conventional and/or molecular methods it was determined that 26.7% (n = 117) of 438 patients were infected with one or more intestinal parasites in this study (Table 1). Protists identified were: *Blastocystis* sp. (16.7%, n = 73), *D*. *fragilis* (11.9%, n = 52), *Giardia duodenalis* (0.7%, n = 3), *Cryptosporidium* spp. (0.7%, n = 3), and *Chilomaxtix mesnili* (0.2%, n = 1). The prevalence of patients positive for these parasites was 37.8% (n = 73) and (24.1%) (n = 59) in immunocompetent and immunocompromised diarrheal patients, respectively(p = 0.001). Information for each protist detected in immunocompetent and immunocompromised diarrheal patients is shown in Table 1.

**Table 1:** Distribution of *Blastocystis* sp., *D*. *fragilis*, and other parasites in immunocompetent and immunodeficient patients with diarrhea.

Protists	Immunodeficient (n = 245)		Immunocompetent (n = 193)		Total (n = 438)	
	N	% (95% CI)	N	% (95% CI)	N	% (95% CI)
*Blastocystis* sp.^a^	31	12.7 (8.9–17.3)	42	21.8 (16.4–28)	73	16.7 (13.4–20.4)
*Dientamoeba fragilis* ^a^	26	10.6 (7.2–14.9)	26	13.5 (9.2–18.9)	52	11.9 (9.1–15.2)
*Giardia duodenalis* ^b^^,^^c^	1	0.4 (0.02–2.0)	2	1.03 (0.2–3.4)	3	0.7 (0.2–1.9)
*Cryprosporidium* spp.^d^	1	0.4 (0.02–2.0)	2	1.03 (0.2–3.4)	3	0.7 (0.2–1.9)
*Chilomastix mesnili* ^b^^,^^c^	-		1	0.5 (0.03–2.5)	1	0.2 (0.01–1.1)
Total	59	24.1 (19–29.7)	73	37.8 (31.2–44.8)	132	30.1 (25.9–34.6)

CI: Confidence Interval

^a^ Data obtained by qPCR.

^b^ Data obtained by direct microscopy.

^c^ Data obtained by trichrome stain.

^d^ Data obtained by modified Kinyoun’s acid-fast stain.

Infection with two or more parasites was reported in 15 (3.4%) patients. The most frequent combination found was *Blastocystis* sp.+*D*. *fragilis* (80.2%, 12/15), followed by *Blastocystis* sp.+*G*. *duodenalis* (6.6%, 1/15), *Blastocystis* sp.+*Cryptosporidium* spp. (6.6%, 1/15) and *Blastocystis* sp.+*C*. *mesnili* (6.6%, 1/15) (S3 Table). The rate of co-infection with *Blastocystis* sp.*+D*. *fragilis* was 4.15% (8/193) in immunocompetent and 1.63% (4/245) in immunodeficient study participants (*χ*^2^ = 2.55, p = 0.11).

### Comparison of methods used in the diagnosis of *Blastocystis* sp. and D. fragilis

The diagnostic performance of saline, Lugol’s iodine, FECT, trichrome stain, and culture were compared with qPCR (Table 2). Both *Blastocystis* sp. and *D*. *fragilis* were detected more frequently by qPCR than by any other detection method.

**Table 2:** Comparison of saline, Lugol’s iodine, formol ethyl acetate concentration technique (FECT), trichrome staining (TS) and culture methods for detecting *Blastocystis* sp. and *D*. *fragilis*. qPCR was used as the reference method for comparing methods by statistical analyses.

	Saline	Lugol’s iodine	FECT	TS	Culture
*Blastocystis* sp. (n/N)	48/438	50/438	46/438	47/438	55/438
Sensitivity	57.3%	61.8%	58.8%	64.7%	77.9%
Specificity	97.6%	97.8%	98.4%	99.2%	99.5%
Positive Predictive Value	81.2%	84.0%	87.0%	93.6%	96.4%
Negative Predictive Value	92.6%	93.3%	92.9%	93.9%	96.1%
Cohen’s kappa (Unweighted)	0.62	0.66	0.66	0.73	0.84
*Dientamoeba fragilis* (n/N)^a^				10/438	
Sensitivity	-	-	-	19.2%	-
Specificity	-	-	-	99%	-
Positive Predictive Value	-	-	-	71.4%	-
Negative Predictive Value	-	-	-	90.1%	-
Cohen’s kappa (Unweighted)				0.27	

^a^ Only TS and qPCR methods were used for detection of *D*. *fragilis*.

When comparing conventional methods with *Blastocystis* sp. qPCR, kappa value was detected as the highest only in the culture method (k = 0.84, perfect agreement). *Dientamoeba fragilis* apart from qPCR could only be detected in trichrome staining and the kappa value was found very low (k = 0.27, fair) (Table 2).

### Occurrence of *Blastocystis* sp. and D. fragilis determined by qPCR

*Blastocystis* sp. was detected using qPCR in 73 patients, including 31 (12.7%) patients with immunodeficiency and 42 (21.8%) immunocompetent patients. The frequency of *Blastocystis* sp. was significantly higher in immunocompetent patients than in immunodeficient patients (*χ*^2^ = 6.40, p = 0.01) (Table 3). Multiple logistic regression analysis revealed that male gender category [*χ*^2^ = 4.78, odds ratio (OR) = 2.33, 95% confidential interval (CI): (1.08,5.03)] and 64–74 age category [*χ*^2^ = 14.43, OR = 8.80, 95% CI: (2.68,28.91)] were risk factors for *Blastocystis* sp. infection among immunocompetent patients (Table 3). With respect to age groups, *Blastocystis* sp. positivity was significantly higher only in the 64–74 age group in immunocompetent patients (*χ*^2^ = 14.43, p<0.001) (Table 3). There were no statistically significant differences in the distribution of total *Blastocystis* sp.-positive patients by season (*χ*^2^ = 1.04, SD = 3, p = 0.79) (Table 3).

**Table 3:** Distribution of the prevalence of *Blastocystis* sp. and *Dientamoeba fragilis* in immunodeficient (n = 245) and immunocompetent patients (n = 193) by gender, age and season (Statistically significant values have been highlighted in bold).

	Immunodeficient patients n/N (%)	Immunocompetent patients n/N (%)	*χ* ^2^	OR (95% CI)	p value^a^
*Blastocystis* sp.	31/245 (12.7)	42/193 (21.8)	6.40	1.92 (1.15–3.19)	**0.01**
Stratified by gender					
Male	12/109 (11.0)	21/94 (22.3)	4.78	2.33 (1.08–5.03)	**0.03**
Female	19/136 (14.0)	21/99 (21.2)	2.10	1.66 (0.84–3.28)	0.15
*χ*^2^ = 1.87, SD = 1, p = 0.17					
Stratified by age category (years)					
18–29	5/31 (16.1)	8/57 (14.0)	0.07	0.85 (0.25–2.86)	0.79
30–40	4/25 (16.0)	5/36 (11.1)	0.05	0.85 (0.20–3.23)	0.82
41–52	6/49 (12.2)	7/31 (22.6)	1.45	2.09 (0.63–6.94)	0.23
53–63	8/68 (11.8)	9/33 (24.2)	3.61	0.36 (0.12–1.03)	0.06
64–74	5/60 (8.33)	12/27 (44.4)	14.43	8.80 (2.68–28.91)	**<0.001**
75–85	3/12 (25.0)	1/9 (11.1)	0.68	0.38 (0.03–4,37)	0.41
*χ*^2^ = 2.14, SD = 5, p = 0.83					
Stratified by seasons category					
Spring	16/97 (16.5)	17/84 (20.2)	0.42	0.78 (0.37–1.66)	0.52
Summer	2/47 (4.2)	5/30 (16.7)	3.34	4.50 (0.81–24.91)	0.06
Autumn	4/52 (7.7)	7/36 (19.4)	2,64	0,35 (0.09–1,28)	0.10
Winter	9/49 (18.3)	13/43 (30.2)	1.77	1.93 (0.73–5.09)	0.19
*χ*^2^ = 1.04, SD = 3, p = 0.79					
*Dientamoeba fragilis*	26/245 (10.6)	26/193 (13.5)	0.84	1.31 (0.73–2.34)	0.36
Stratified by gender					
Male	11/109 (10.1)	11/94 (11.7)	0.14	1.18 (0.49–2.86)	0.71
Female	15/136 (11.0)	15/99 (15.2)	0.86	1.44 (0.67–3.10)	0.35
*χ*^2^ = 0, SD = 1, p = 0.99					
Stratified by age category (years)					
18–29	5/31 (16.1)	6/57 (10.5)	0.56	0.61 (0.17–2.19)	0.45
30–40	3/25 (12.0)	7/36 (19.4)	0.62	1.77 (0.41–7.63)	0.44
41–52	7/49 (14.3)	4/31 (12.9)	0.03	0.89 (0.24–3.33)	0.86
53–63	4/68 (5.9)	3/33 (9.1)	0.34	1.60 (0.34–7.60)	0.55
64–74	7/60 (11.7)	4/27 (14.8)	0.16	1.32 (0.35–4.94)	0.68
75–85	-/12 (-)	2/9 (22.2)	3.67		
*χ*^2^ = 5.47, SD = 5, p = 0.36					
Stratified by seasons category					
Spring	17/97 (17.5)	10/84 (11.9)	1.14	0.64 (0.27–1.48)	0.29
Summer	2/47 (4.3)	2/30 (6.7)	0.21	1.61 (0.21–12.06)	0.64
Autumn	3/52 (5.8)	6/36 (16.7)	2.70	3.27 (0.76–14.04)	0.11
Winter	4/49 (8.2)	8/43 (18.6)	2.22	2.57 (0.72–9.24)	0.14
*χ*^2^ = 4.15, SD = 3, p = 0.25					

^a^ Multiple logistic regression analysis

Cycle threshold (Ct) values of *Blastocystis* sp.-positive patients with immunodeficiency ranged from 16.7 to 34.6 (median: 21.6), and immunocompetent patients ranged from 17 to 34.8 (median: 24.9). The median of *Blastocystis* sp. Ct values was found to be lower in women with immunodeficiency compared to immunocompetent women, indicating a higher parasite load in immunodeficient women (p = 0.04). However, differences of the median *Blastocystis* sp. Ct values were not found statistically significant between immunodeficient (median: 21.6) and immunocompetent (median:24.9) males (p = 0.40) (Table 4).

**Table 4:** Median of qPCR cycle threshold (Ct) values of *Blastocystis* sp. and *D*. *fragilis* (Statistically significant values have been highlighted in bold).

	*Blastocystis* sp.			*Dientamoeba fragilis*		
	Immuno deficient	Immuno competent	p^a^	Immuno deficient	Immuno competent	p^a^
Gender						
Male	26.7	23.5	0.26	32.0	32.2	0.69
Female	21.4	26.5	**0.04**	31.4	32.1	0.83
Age						
18–40	20.2	25.2	0.42	30.8	32.2	0.55
41–63	21.5	26.5	0.16	31.6	33.1	0.96
>64	25.2	23.5	0.35	31.6	25.1	0.07
Total	21.6	24.9	0.40	31.6	32.2	0.53

^a^T-test for Independent Samples

*Dientamoeba fragilis* was detected using qPCR in 52 patients, including 26 (11.4%) patients with immunodeficiency and 26 (20.7%) immunocompetent patients. The frequency of *D*. *fragilis* between the two groups was not significantly different (*χ*^2^ = 0,84, p = 0.36) (Table 3). Similar Ct values were found in *D*. *fragilis*-positive patients with immunodeficiency (16.5 to 34.4; median: 31.6) and in immunocompetent patients (15.1 to 34.7; median: 32.2) (p = 0.53) (Table 4).

### *Blastocystis* sp. subtypes identified using NGS

Out of the 73 positive qPCR samples, only 68 isolates were positive when PCR was done to prepare NGS library. The remaining five samples were not sequenced using the MiSeq platform. Clustering yielded 40 unique *Blastocystis* sp. OTUs across the 68 *Blastocystis* sp.-positive samples (Table 5). Five *Blastocystis* sp. subtypes (ST1, ST2, ST3, ST4, and ST6) were found. ST4 was detected only in an immunocompetent patient.

**Table 5:** *Blastocystis* sp. subtypes identified by next generation sequencing including information about number of variants per subtype and patients ID in which they were found. Bold denotes intra-subtype variability.

Subtype	No. of unique subtype variants	GenBank Accession number	No. of samples containing variant	Patients ID^1^
ST1	8	MW728059	3	**ID/F42,** IY/F82, IY/F114
		MW728064	3	ID/F18, ID/F91, ID/F99
		MW728065	1	**ID/F184**
		MW728079	1	IY/F78
		MW728086	1	**ID/F165**
		MW728088	1	**ID/F165**
		MW728091	1	**ID/F42**
		MW728092	1	**ID/F184**
ST2	20	MW728061	6	**ID/F68**, **ID/F104**, **ID/F157**, **ID/F170**, IY/F124, **IY/F180**,
		MW728078	4	**ID/F16**, **ID/F100**, **IY/F34**, **IY/F109**
		MW728066	2	ID/F87, ID/F150
		MW728070	2	**ID/F68**, **ID/F170**
		MW728072	2	**ID/F16**, **IY/F5**
		MW728076	2	**ID/F125**, **IY/F108**
		MW728083	2	**ID/F125**, **IY/F5**
		MW728067	1	ID/F180
		MW728068	1	**ID/F173**
		MW728071	1	**ID/F104**
		MW728075	1	IY/F185
		MW728077	1	**IY/F34**
		MW728080	1	**IY/F109**
		MW728082	1	**ID/F157**
		MW728084	1	**IY/F108**
		MW728084	1	**IY/F180**
		MW728087	1	**ID/F100**
		MW728089	1	**IY/F109**
		MW728090	1	**ID/F173**
		MW728093	1	ID/F43
ST3	9	MW728054	27	ID/F18, ID/F33, ID/F42, ID/F43, ID/F47, ID/F71, ID/F85, ID/F89, ID/F99, ID/F100, ID/F158, ID/F164, ID/F169, IY/F1, IY/F6, IY/F34, IY/F59, IY/F82, IY/F85, IY/F100, IY/F115, IY/F121, IY/F133, IY/F170, IY/F206, ID/F165, ID/F170
		MW728055	6	ID/F115, ID/F133, ID/F188, ID/F204, IY/F78, IY/F156
		MW728056	6	ID/F37, ID/F38, ID/F72, ID/F87, IY/F136, IY/F171
		MW728057	4	ID/F16, ID/F45, IY/F26, IY/F74
		MW728058	3	ID/F67, ID/F68, ID/F129
		MW728060	2	ID/F159, ID/F185
		MW728062	1	ID/F210
		MW728063	1	ID/F63
		MW728081	1	IY/F180
ST4	1	MW728074	1	ID/F151
ST6	2	MW728069	1	ID/F109
		MW728073	1	IY/F174

ID/F: Immunocompetent patient group; IY/F: Immunodeficient patient group.

Mono-subtype infections were more common than mixed infections representing 79.4% (n = 54) and 20.6% (n = 14) of the positive samples, respectively (Tables 6 and S4). ST3 was observed in 54.4% (n = 37) of *Blastocystis* sp. positive patients and was the most common subtype observed. The prevalence of other subtypes was as follows: ST2: 16.2% (n = 11), ST1: 4.4% (n = 3), ST6: 2.9% (n = 2), and ST4: 1.5% (n = 1) (Table 6). While mixed subtypes were higher in immunocompetent patients (25.0%) than in immunodeficient patients (14.2%) the difference was not statistically significant. Mixed ST2/ST3 and ST1/ST3 were observed with ST2/ST3 (11.8%) being the most common subtype combination (Table 6).

**Table 6:** Distribution of *Blastocystis* sp. subtypes in the immunodeficient (n = 28) and immunocompetent (n = 40) patient groups.

Subtype	Number immunodeficient patients (%)	Number of immunocompetent patients (%)	Total number of patients (%)	*χ* ^2^	p^a^
ST1	1 (3.6)	2 (5)	3 (4.4)	0.07	0.78
ST2	6 (21.4)	5 (12.5)	11 (16.2)	0.97	0.33
ST3	16 (57.2)	21 (52.5)	37 (54.4)	0.14	0.71
ST4	-	1 (2.5)	1 (1.5)	0.71	0.39
ST6	1 (3.6)	1 (2.5)	2 (2.9)	0.07	0.80
Total mixed subtypes	4 (14.2)	10 (25.0)	14 (20.6)	1.16	0.28
ST1/ST3	2 (7.1)	4 (10.0)	6 (8.8)	0.17	0.68
ST2/ST3	2 (7.1)	6 (15.0)	8 (11.8)	0.98	0.32

^a^ Statistical analysis using Chi-Square Test for two-way tables.

### *Blastocystis* sp. intra-subtype variability

Forty unique OTUs were detected among the five *Blastocystis* sp. subtypes identified in this study. A high degree of intra-subtype diversity was observed for ST1 and ST2 with eight unique OTUs among the nine ST1-positive samples and 20 unique OTUs among the 19 ST2-positive samples (Table 5). ST1 and ST2 *Blastocystis* sp. positive samples frequently contained multiple unique OTUs. In fact, up to three unique OTUs were detected in a single ST2 sample (Table 5). Interestingly, ST3 displayed low intra-subtype diversity relative to the number of positive isolates, with only nine unique OTUs among 51 ST3-positive samples. ST4 had one unique OTU in only one ST4-positive sample. ST6 had two unique OTUs among two ST6-positive samples.

## Discussion

In the present study, *Blastocystis* sp. and *D*. *fragilis* were investigated in immunodeficient and immunocompetent diarrheal patients using conventional and molecular methods. This study demonstrates that the successful diagnosis of *Blastocystis* sp. and *D*. *fragilis* infections depends on the detection method. Using conventional methods such as direct smear, it is possible to overlook protists, especially when few organisms are present. In fact, *D*. *fragilis* was not detected in any samples by direct smear. In the diagnosis of *Blastocystis* sp., the sensitivity of direct smear, FECT, and trichrome smear was significantly lower than qPCR, while culture had a similar diagnostic accuracy when compared to qPCR. Our findings are in agreement with other studies that have also shown low sensitivity of microscopic methods to detect *Blastocystis* sp. and *D*. *fragilis* [56, 74]. Studies that compared direct microscopy, culture and qPCR for *Blastocystis* sp. detection have reported that qPCR was the most sensitive method [75, 76]. Similarly, a study comparing just culture and qPCR to detect *Blastocystis* sp. reported a higher sensitivity of qPCR [67]. However, the effectiveness of the culture method for *Blastocystis* sp. diagnosis has also been demonstrated by other researchers [77, 78] and the mini-culture method is a practical method especially for diagnostic laboratories with a limited budget [79].

In this study, the overall prevalence of *Blastocystis* sp. was 16.7% (n = 73/438) by qPCR. The prevalence of *Blastocystis* sp. has been reported to vary widely among studies ranging from 0.54% to 88.8% [20, 26, 47, 80–82]. Prevalence variations could be related to many factors such as studies conducted in different geographical regions, different populations (socio-economic level, immune status, age…), or use of different diagnostic methods for detection [34]. In Turkey, *Blastocystis* sp. is the most common gastrointestinal parasite reported and prevalence ranges between 0.5% to 37.9% (S5 Table) [18, 21, 83–90]. *Blastocystis* sp. carriage was detected in limited studies from the same province in Turkey as 14.2–14.6% in school children [91, 92] and 15.5% in adults [75]. Both the present and the other studies from Turkey indicate no apparent differences between symptomatic and asymptomatic cases, supporting the commensal nature of *Blastocystis* sp. [75, 91, 92].

*Blastocystis* sp. was observed in 20.7% and 11.4% of immunocompetent and immunodeficient diarrheal patients examined in this study, respectively. One limitation of this study is a lack of a healthy comparison group (no diarrhea) as all samples were collected from hospital patients presenting with diarrhea. Thus, no comparison can be made of *Blastocysits* prevalence between symptomatic and asymptomatic individuals. The prevalence of *Blastocystis* sp. observed in immunodeficient patients in this study is similar to prevalence reported in cancer patients in Turkey (6.5–10.8%) (S5 Table) [84, 93]. However, studies in some neighbour countries have reported higher *Blastocystis* sp. prevalence in patients with cancer ranging from 22.3 to 27.5% [14, 94]. In a prospective study in France using qPCR, similar prevalence of *Blastocystis* sp. was found in immunodeficient patients (16%;15/94) and immunocompetent patients (13%;12/92) [76]. In our study, immunocompetent group had a significantly higher *Blastocystis* sp. prevalence than immunocompromised group. It is possible that immunocompromised patients that are under constant medical monitoring tend also to avoid contact with the external environment and behave carefully making them less likely to contract parasitic infections.

We did not observe significant differences in *Blastocystis* sp. prevalence between males and females. However, a previous study detected *Blastocystis* sp. more frequently in males than in females in immunocompromised patients in Iran [95, 96]. We found significant differences in *Blastocystis* sp. prevalence between immunocompetent males (22.3%) and immunodeficient males (11.0%). Additionally, a significantly higher *Blastocystis* sp. infection rate was observed in the age range of 64–74 years in immunocompetent patients (44.4%) than in immunocompromised patients (8.3%). This differs from results of a study conducted in Turkey in patients with gastrointestinal symptoms that found highest prevalence of *Blastocystis* sp. in the 20–29 age group (28.9%) [75]. Unfortunately, there was no information regarding to the immune statuses of those patients. An age-related epidemiological pattern was also reported in a study in France in which higher prevalence was observed in patients between 15 and 49 years of age (22.2%) than the patients over 50 years of age (16.6%) [26]. In the same study, the prevalence of *Blastocystis* sp. in immunocompromised subjects (12.4%) was significantly lower than in immunocompetent patients (24.2%). The patients were further divided into immunocompromised subgroups which were HIV, solid organ transplants, immunosuppressive therapy, solid cancer, and bone marrow transplants. The results of these subgroup analyses validated that the prevalence of *Blastocystis* sp. was considerably lower in subjects getting immunosuppressive treatment (8.4%) and bone marrow transplant (7.7%) but not significantly lower in the other subgroups [26]. The patients recieving bone marrow transplant had lower prevalence (7.1%) than the other immunodeficient patient subgroups (S1 Table).

Because qPCR allows quantification, it has been reported that the use of qPCR in large-scale surveys could assist in identifying whether the development of symptoms is related to infection intensity by simple analysis of Ct values [34]. We found a significantly lower median Ct value in immunocompromised females (Ct = 21.4) than in immunocompetent females (Ct = 26.5) (Table 4) that appears to indicate a higher burden of *Blastocystis* sp. in females with immunodeficiency. A multicenter case-control study in The Netherlands designed to clarify the clinical importance of qPCR in patients with gastroenteritis found a higher mean (SD) Ct value of *Blastocystis* sp. and *D*. *fragilis* in cases as compared to controls [6]. Interestingly, the hypothesis of this study was that during an episode of diarrhea, “a flush out effect” could decrease the load of *Blastocystis* sp.. However, no information was given about the immune status of the cases.

*Dientamoeba fragilis* prevalence has been reported to range from 0% to 82.9% in studies using conventional or molecular methods [12, 43, 97]. Differences have been attributed to variations associated to geographical region, socioeconomic status, and diagnostic methods [12, 51]. In Turkey, the prevalence of *D*. *fragilis* ranges from 0% to 18.3% [48, 49, 97–109] (S6 Table). The overall prevalence of *D*. *fragilis* found in this study (11.9%) falls within the previously reported range for Turkey. A limited number of studies exist on *D*. *fragilis* carriage in Turkey. However, *D*. *fragilis* was found in 3.4% of school children by trichrome staining in one study [91]. In another study, *D*. *fragilis* was detected in 12.04% of outpatients with gastrointestinal symptoms such as diarrhea, abdominal pain and nausea [48]. In the second study, real-time PCR was used and diarrhea was statistically more significant in patients with the presence of *D*. *fragilis*. In this study, although *D*. *fragilis* was detected more frequently (13.5%) in the immunocompetent patients with diarrhea than immunodeficient patients with diarrhea (10.6%), these differences were not statistically significant. In Turkey, using qPCR, two studies reported a prevalence for *D*. *fragilis* in patients with gastrointestinal symptoms, with unknown immune status, of 10.7% and 12.4% [48, 109]. There are only a few articles about the prevalence of *D*. *fragilis* in immunodeficient patients. In a study from Iran, *D*. *fragilis* was found 1.2% of 190 patients including primary immunodeficiency patients, cancer patients and organ transplants recipients [14]. A study in HIV positive men with diarrhea in Australia reported a prevalence of 0.3% for *D*. *fragilis* [44]. *Dientamoeba fragilis* was detected only by microscopy in both studies. In the Netherlands, using the multiplex real-time PCR method *D*. *fragilis* was detected in 25.8% and 37.6% of patients with gastrointestinal complaints and without complaints, respectively [6]. In our study, no statistically significant differences were found between the two groups in terms of prevalence, gender, age, and seasonal variables for *D*. *fragilis* (Table 3). Case-control studies with a large number of samples are needed to determine the relationship between *D*. *fragilis* and symptomatology.

In this study, co-infection with *D*. *fragilis* was detected in 12 (17.6%) of the of 73 *Blastocystis* sp. positives. This coinfection rate was lower than previously reported in other studies [48, 95, 110]. To investigate the relationship between *D*. *fragilis* colonization and specific gastrointestinal symptoms, and sociodemographic characteristic, a cross-sectional study that included 490 fecal specimens were collected from outpatient with gastrointestinal symptoms and tested with qPCR in Turkey [48]. Their results suggested that *D*. *fragilis* is a pathogenic parasite and that the most common clinical symptom found in infected patients is diarrhea [48]. They found that 23.7% were co-infected with *Blastocystis* sp. [48]. A study in Italy that included 756 patients suspected of harboring intestinal parasites were subjected to multiplex RT-PCRs to detect parasites [95]. The prevalance of *Blastocytis* was 34% and co-infection with *D*. *fragilis* was detected in 24% of patients [95]. Our study showed lower co-infection than both of those studies. The common occurrence of co-infection of these two parasites may indicate that there are shared sources of transmission.

Low infection rates were found for *G*. *duodenalis* and *Cryptosporidium* spp. in this study (<1%). This situation may be explained in part by the study location which is located in the center of Ankara, the second biggest city and the capital city of Turkey. Ankara is known also as a civil servant city, far from the places where agriculture and animal husbandry are made. The majority of the population lives in apartments and uses carboys or purified water. Healthcare workers like nurses and doctors also inform immunocompromised patients about protection from infections. Similar results were obtained in a study conducted in another university hospital in Ankara. The period of the study was 2003–2012 and *G*. *duodenalis* and *Crptosporidium* spp. prevalences were 1.3% and 0.003% respectively. Out of the 85,707 fecal samples examined, 3,681 (4.2%) were positive to parasites [108]. Another study also reported low prevalence of *G*. *duodenalis* (0.61%) and *Cryptosporidium* spp. (0%) from one of university hospitals in Izmir city (the third biggest city of Turkey) in the western region of Turkey [48]. To date, several methods based on specific primers for determining subtypes of *Blastocystis* sp. have been developed [60, 70, 111–115]. However, only a few of these methods can be used to determine mixed subtypes [111, 113, 115, 116]. The disadvantages of STS-PCR [113, 116] and ST-specific nested PCR assay [115] are being time-consuming, having a high risk of contamination, and being able to identify only certain STs. Recently, NGS was used for identification of *Blastocystis* sp. subtypes with the advantage of allowing identification of all currently known subtypes, the ability to evaluate mixed infections, and the ability to detect intra-subtype diversity within a single sample quickly and with only a small amount of input material conserving both time and valuable sample material. [8, 30]. Furthermore, the NGS method provides improved sensitivity for identifying subtypes with zoonotic potential that could be in low proportions within a specimen. This is the first study adopting an NGS method to investigate the genetic diversity of *Blastocystis* sp. in Turkey, as previous studies conducted in the country were based on STS-PCR and Sanger sequencing *[21, 83–87]*. NGS was used to study *Blastocystis* sp. genetic diversity in a rural human population from Mexico where 3 subtypes were reported, ST1-ST3, with the following frequencies: ST3 (67.7%), (ST2 11.3%), ST1 (7.3%), ST1/ST3 (7.3%), ST2/ST3 (4.0%), and ST1/ST2/ST3 (2.4%) [8]. Both the present study and the study in Mexico report ST1, ST2, and ST3, with ST3 being the most prevalent subtype. However, we have a higher proportion of mixed infections than the study in Mexico. Another difference between the two studies is that we identified two additional subtypes in patients in Turkey, ST4 and ST6. Previous molecular studies in Turkey have shown that ST1-ST4 represent 87.5% of *Blastocystis* sp. positive human samples, ST5-ST7 represent 3.5%, and mixed subtype infections represent 9% (S5 and S7 Tables).

Globally, ST1-ST4 have been identified as the most common subtypes in humans. Thus, the findings of our study are consistent with other studies carried out in Turkey and worldwide [10, 75, 83, 87, 117, 118]. ST2 was the second most common ST in our study. This is in agreement with other studies in humans that also report ST2 as the second most common ST after ST3 [8, 81, 119–121]. In Turkey, Dogruman-Al, et al. (2008) reported ST2 as the second most common subtype after ST3 with a statistically significant association to asymptomatic patients, suggesting that ST2 may be a non-pathogenic subtype of *Blastocystis* sp. in this population [117]. Another study in Turkey reported a relationship between ST1 and abdominal pain [122]. In our study, ST4, which is generally found across Europe, was detected in only one immunocompetent patient. In Turkey, reports in humans of ST4 are sporadic (S4 Table) [86, 118, 123]. In our study, ST6 was detected in one immunocompetent and one immunodeficient patient. There are other reports of ST6 in humans from Turkey, Poland and South America [30, 31, 33, 75, 87, 123, 124]. Because ST6 is mostly identified in birds, its presence in humans may indicate the potential for zoonotic transmission [31]. In the present study, mixed subtype infections of *Blastocystis* sp. represented 20.6% of the total subtype identified. The incidence of mixed subtypes in Turkey has been reported to range from 3.2% to 30.5% with the STS-PCR method (S7 Table). We found no statistically significant differences between immunocompetent and immunodeficient patients for both *Blastocystis* sp. positivity and mixed subtype distribution (Table 6). The importance of the mixed subtype infection of *Blastocystis* sp. is not clear yet for symptomatology or pathogenicity, and additional studies that include methodology to detect mixed subtype infections are needed.

Some researchers have suggested that intra-subtype variability could have a role in the transmission and pathogenicity of *Blastocystis* sp. [8, 29, 30, 64, 125]. This study showed intra-subtype variations only for ST2 and ST1. Other subtypes (ST3, ST4, and ST6) did not have within sample intra-subtype variations. However, result for ST4 and ST6 should be taken with caution because ST4 and ST6 were only identified in 1, and 2 patients, respectively. Intra-subtype variability observed for ST1-ST3 is consistent with intra-subtype variations reported in previous studies in Iran and Mexico [8, 126]. In the study in Mexico, intra-subtype variability was also more common in ST2 and ST1 than in ST3 [8]. However, there is still limited data and more studies reporting intra-subtype variability are needed to understand the potential role of this variability in pathogenicity, zoonotic potential, and transmission.

This study provides valuable information about *Blastocystis* sp. and *D*. *fragilis* in humans. However, because no healthy controls were available for comparison, conclusions about the role of these parasites in health and disease could not be drawn. In the future, further case-control studies implementing high-resolution molecular tools or functional genomic analysis are necessary to understand the role of intra-subtype variation of *Blastocystis* sp. in pathogenicity or symptomatology and the role of neglected protists in health and disease.

## Supporting information

S1 Table Distribution of immunocompetent and immunodeficient patients according to clinics and protist. (DOCX)

S2 Table Oligonucleotides which were used for the molecular identification and/or characterization of *Blastocystis* sp., and *Dientamoeba fragilis* in the present study. (DOCX)

S3 Table Coinfections (n = 15) with other enteric parasitic and commensal species detected in the investigated patients. (DOCX)

S4 Table *Blastocystis* sp. subtypes relative abundance in positive samples identified by next generation sequencing. (DOCX)

S5 Table *Blastocystis* sp. carriage rates and subtype diversity reported in human samples in Turkey during the period 2000–2019. (DOCX)

S6 Table *Dientamoeba fragilis* prevalence in human samples in Turkey. (DOCX)

S7 Table *Blastocystis* sp. subtype diversity in Turkish human populations based on the molecular data. (DOCX)

## References

[1] Krones E, Högenauer C. Diarrhea in the immunocompromised patient. Gastroenterol Clin North Am. 2012;41(3):677–701. doi: 10.1016/j.gtc.2012.06.009 22917171

[2] Törnblom H, Holmvall P, Svenungsson B, Lindberg G. Gastrointestinal symptoms after infectious diarrhea: a five-year follow-up in a Swedish cohort of adults. Clin Gastroenterol Hepatol. 2007;5(4):461–464. doi: 10.1016/j.cgh.2007.01.007 17445752

[3] Svenungsson B, Lagergren A, Ekwall E, Evengård B, Hedlund KO, Kärnell A, et al. Enteropathogens in adult patients with diarrhea and healthy control subjects: a 1-year prospective study in a Swedish clinic for infectious diseases. Clin Infect Dis. 2000;30(5):770–778. doi: 10.1086/313770 10816147

[4] Dubey JP, Almeria S. *Cystoisospora belli* infections in humans: the past 100 years. Parasitology. 2019;146(12):1490–1527. doi: 10.1017/S0031182019000957 31303182

[5] Laksemi DA, Suwanti LT, Mufasirin M, Suastika K, Sudarmaja M. Opportunistic parasitic infections in patients with human immunodeficiency virus/acquired immunodeficiency syndrome: A review. Vet World. 2019;13(4):716–725. doi: 10.14202/vetworld.2020.716-725 32546916 PMC7245710

[6] de Boer MD, Schuurs TA, Vermeer M, Ruijs GJHM, van der Zanden AGM, Weel JF, et al. Distribution and relevance of *Dientamoeba fragilis* and *Blastocystis* species in gastroenteritis: results from a case-control study. Eur J Clin Microbiol Infect Dis. 2020;39(1):197–203. doi: 10.1007/s10096-019-03710-z 31659566

[7] Stensvold CR, Clark CG. Pre-empting Pandora’s Box: *Blastocystis* subtypes revisited. Trends Parasitol. 2020;36(3):229–232. doi: 10.1016/j.pt.2019.12.009 32001133

[8] Rojas-Velázquez L, Maloney JG, Molokin A, Morán P, Serrano-Vázquez A, González E, et al. Use of next-generation amplicon sequencing to study *Blastocystis* genetic diversity in a rural human population from Mexico. Parasit Vectors. 2019;12(1):566. doi: 10.1186/s13071-019-3814-z 31775832 PMC6882168

[9] Barratt JL, Harkness J, Marriott D, Ellis JT, Stark D. A review of *Dientamoeba fragilis* carriage in humans: several reasons why this organism should be considered in the diagnosis of gastrointestinal illness. Gut Microbes. 2011;2(1):3–12. doi: 10.4161/gmic.2.1.14755 21637013

[10] Dogruman-Al F, Kustimur S, Yoshikawa H, Tuncer C, Simsek Z, Tanyuksel M, et al. *Blastocystis* subtypes in irritable bowel syndrome and inflammatory bowel disease in Ankara, Turkey. Mem Inst Oswaldo Cruz. 2009;104(5):724–727. doi: 10.1590/s0074-02762009000500011 19820833

[11] Ramírez JD, Sánchez LV, Bautista DC, Corredor AF, Flórez AC, Stensvold CR. *Blastocystis* subtypes detected in humans and animals from Colombia. Infect Genet Evol. 2014;22:223–228. doi: 10.1016/j.meegid.2013.07.020 23886615

[12] Stark D, Barratt J, Chan D, Ellis JT. *Dientamoeba fragilis*, the neglected trichomonad of the human bowel. Clin Microbiol Rev. 2016;29(3):553–580. doi: 10.1128/CMR.00076-15 27170141 PMC4861990

[13] Stenzel DJ, Boreham PF. *Blastocystis hominis* revisited. Clin Microbiol Rev. 1996;9(4):563–584. doi: 10.1128/CMR.9.4.563 8894352 PMC172910

[14] Esteghamati A, Khanaliha K, Bokharaei-Salim F, Sayyahfar S, Ghaderipour M. Prevalence of intestinal parasitic infection in cancer, organ transplant and primary immunodeficiency patients in Tehran, Iran. Asian Pac J Cancer Prev. 2019;20(2):495–501. doi: 10.31557/APJCP.2019.20.2.495 30803212 PMC6897035

[15] Fontanelli Sulekova L, Gabrielli S, Furzi F, Milardi GL, Biliotti E, De Angelis M, et al. Molecular characterization of *Blastocystis* subtypes in HIV-positive patients and evaluation of risk factors for colonization. BMC Infect Dis. 2019;19(1):876. doi: 10.1186/s12879-019-4537-7 31640585 PMC6805496

[16] Andersen LO, Stensvold CR. *Blastocystis* in health and disease: Are we moving from a clinical to a public health perspective?. J Clin Microbiol. 2016;54(3):524–528. doi: 10.1128/JCM.02520-15 26677249 PMC4767957

[17] Belleza ML, Reyes JC, Tongol-Rivera PN, Rivera WL. Subtype analysis of *Blastocystis* sp. isolates from human and canine hosts in an urban community in the Philippines. Parasitol Int. 2016;65(3):291–294. doi: 10.1016/j.parint.2016.02.009 26902433

[18] Beyhan YE, Yilmaz H, Cengiz ZT, Ekici A. Clinical significance and prevalence of *Blastocystis hominis* in Van, Turkey. Saudi Med J. 2015;36(9):1118–1121. doi: 10.15537/smj.2015.9.12444 26318472 PMC4613639

[19] El Safadi D, Gaayeb L, Meloni D, Cian A, Poirier P, Wawrzyniak I, et al. Children of Senegal River Basin show the highest prevalence of *Blastocystis* sp. ever observed worldwide. BMC Infect Dis. 2014;14:164. doi: 10.1186/1471-2334-14-164 24666632 PMC3987649

[20] Sánchez A, Munoz M, Gómez N, Tabares J, Segura L, Salazar Á, et al. Molecular Epidemiology of *Giardia*, *Blastocystis* and *Cryptosporidium* among Indigenous Children from the Colombian Amazon Basin. Front Microbiol. 2017;8:248. doi: 10.3389/fmicb.2017.00248 28270802 PMC5318379

[21] Sankur F, Ayturan S, Malatyali E, Ertabaklar H, Ertug S. The distribution of *Blastocystis* subtypes among school-aged children in Mugla, Turkey. Iran J Parasitol. 2017;12(4):580–586. 29317883 PMC5756308

[22] Seguí R, Muñoz-Antoli C, Klisiowicz DR, Oishi CY, Köster PC, De Lucio A, et al. Prevalence of intestinal parasites, with emphasis on the molecular epidemiology of *Giardia duodenalis* and *Blastocystis* sp., in the Paranaguá Bay, Brazil: a community survey. Parasit Vectors. 2018;11(1):490. doi: 10.1186/s13071-018-3054-7 30165880 PMC6117969

[23] Zhang W, Ren G, Zhao W, Yang Z, Shen Y, Sun Y, et al. Genotyping of *Enterocytozoon bieneusi* and subtyping of *Blastocystis* in cancer patients: Relationship to diarrhea and assessment of zoonotic transmission. Front Microbiol. 2017;8:1835. doi: 10.3389/fmicb.2017.01835 28983297 PMC5613175

[24] Bartolini A, Zorzi G, Besutti V. Prevalence of intestinal parasitoses detected in Padua teaching hospital, Italy, March 2011—February 2013. Infez Med. 2017;25(2):133–141. 28603232

[25] Bednarska M, Jankowska I, Pawelas A, Piwczyńska K, Bajer A, Wolska-Kuśnierz B, et al. Prevalence of *Cryptosporidium*, *Blastocystis*, and other opportunistic infections in patients with primary and acquired immunodeficiency. Parasitol Res. 2018;117(9):2869–2879. doi: 10.1007/s00436-018-5976-6 29946765 PMC6105259

[26] El Safadi D, Cian A, Nourrisson C, Pereira B, Morelle C, Bastien P, et al. Prevalence, risk factors for infection and subtype distribution of the intestinal parasite *Blastocystis* sp. from a large-scale multi-center study in France. BMC Infect Dis. 2016;16(1):451. doi: 10.1186/s12879-016-1776-8 27566417 PMC5002209

[27] Paulos S, Köster PC, De Lucio A, Hernández-De-Mingo M, Cardona GA, Fernández-Crespo JC, et al. Occurrence and subtype distribution of *Blastocystis* sp. in humans, dogs and cats sharing household in northern Spain and assessment of zoonotic transmission risk. Zoonoses Public Health. 2018;65(8):993–1002. doi: 10.1111/zph.12522 30198123

[28] Scanlan PD, Knight R, Song SJ, Ackermann G, Cotter PD. Prevalence and genetic diversity of *Blastocystis* in family units living in the United States. Infect Genet Evol. 2016;45:95–97. doi: 10.1016/j.meegid.2016.08.018 27545648

[29] Maloney JG, Lombard JE, Urie NJ, Shivley CB, Santin M. Zoonotic and genetically diverse subtypes of *Blastocystis* in US pre-weaned dairy heifer calves. Parasitol Res. 2019;118(2):575–582. doi: 10.1007/s00436-018-6149-3 30483890

[30] Maloney JG, Molokin A, da Cunha MJR, Cury MC, Santin M. *Blastocystis* subtype distribution in domestic and captive wild bird species from Brazil using next generation amplicon sequencing. Parasite Epidemiol Control. 2020;9:e00138. doi: 10.1016/j.parepi.2020.e00138 32021915 PMC6995250

[31] Hublin JSY, Maloney JG, Santin M. *Blastocystis* in domesticated and wild mammals and birds. Res Vet Sci. 2020;S0034-5288(20)31032-8. doi: 10.1016/j.rvsc.2020.09.031 33046256

[32] Stensvold CR, Tan KSW, Clark CG. *Blastocystis*. Trends Parasitol. 2020;36(3):315–316. doi: 10.1016/j.pt.2019.12.008 32001134

[33] Ramírez JD, Sánchez A, Hernández C, Flórez C, Bernal MC, Giraldo JC, et al. Geographic distribution of human *Blastocystis* subtypes in South America. Infect Genet Evol. 2016;41:32–35. doi: 10.1016/j.meegid.2016.03.017 27034056

[34] Stensvold CR, Clark CG. Current status of *Blastocystis*: A personal view. Parasitol Int. 2016;65(6 Pt B):763–771. doi: 10.1016/j.parint.2016.05.015 27247124

[35] Mattiucci S, Crisafi B, Gabrielli S, Paoletti M, Cancrini G. Molecular epidemiology and genetic diversity of *Blastocystis* infection in humans in Italy. Epidemiol Infect. 2016;144(3):635–646. doi: 10.1017/S0950268815001697 26194649

[36] Khaled S, Gantois N, Ly AT, Senghor S, Even G, Dautel E, et al. Prevalence and Subtype Distribution of *Blastocystis* sp. in Senegalese School Children. Microorganisms. 2020;8(9):1408. doi: 10.3390/microorganisms8091408 32932661 PMC7564003

[37] Aynur ZE, Güçlü Ö, Yıldız İ, Aynur H, Ertabaklar H, Bozdoğan B, et al. Molecular characterization of *Blastocystis* in cattle in Turkey. Parasitol Res. 2019;118(3):1055–1059. doi: 10.1007/s00436-019-06243-8 30739165

[38] Greige S, El Safadi D, Khaled S, Gantois N, Baydoun M, Chemaly M, et al. First report on the prevalence and subtype distribution of *Blastocystis* sp. in dairy cattle in Lebanon and assessment of zoonotic transmission. Acta Trop. 2019;194:23–29. doi: 10.1016/j.actatropica.2019.02.013 30878470

[39] Stensvold CR. *Blastocystis*: Genetic diversity and molecular methods for diagnosis and epidemiology. Trop Parasitol. 2013;3(1):26–34. doi: 10.4103/2229-5070.113896 23961438 PMC3745667

[40] Brands MR, Van de Vijver E, Haisma SM, Heida A, van Rheenen PF. No association between abdominal pain and *Dientamoeba* in Dutch and Belgian children. Arch Dis Child. 2019;104(7):686–689. doi: 10.1136/archdischild-2018-316383 30798256 PMC6589455

[41] Crotti D, D’Annibale ML. [Role of *Dientamoeba fragilis* in human bowel infections]. Infez Med. 2007;15(1):30–39. Italian. 17515673

[42] Pietilä J-P, Meri T, Siikamäki H, Tyyni E, Kerttula A-M, Pakarinen L, et al. *Dientamoeba fragilis*—the most common intestinal protozoan in the Helsinki Metropolitan Area, Finland, 2007 to 2017. Euro Surveill. 2019;24(29):1800546. 10.2807/1560-7917.ES.2019.24.29.1800546 PMC6652114 31339096

[43] Preiss U, Ockert G, Brömme S, Otto A. *Dientamoeba fragilis* infection, a cause of gastrointestinal symptoms in childhood. Klin Padiatr. 1990;202(2):120–123. doi: 10.1055/s-2007-1025503 2325352

[44] Stark D, Fotedar R, van Hal S, Beebe N, Marriott D, Ellis JT, et al. Prevalence of enteric protozoa in human immunodeficiency virus (HIV)-positive and HIV-negative men who have sex with men from Sydney, Australia. Am J Trop Med Hyg. 2007;76(3):549–552. 17360882

[45] Greigert V, Abou-Bacar A, Brunet J, Nourrisson C, Pfaff AW, Benarbia L, et al. Human intestinal parasites in Mahajanga, Madagascar: The kingdom of the protozoa. PLoS One. 2018;13(10):e0204576. doi: 10.1371/journal.pone.0204576 30304028 PMC6179227

[46] Jimenez-Gonzalez DE, Martinez-Flores WA, Reyes-Gordillo J, Ramirez-Miranda ME, Arroyo-Escalante S, Romero-Valdovinos M, et al. *Blastocystis* infection is associated with irritable bowel syndrome in a Mexican patient population. Parasitol Res. 2012;110(3):1269–1275. doi: 10.1007/s00436-011-2626-7 21870243

[47] Osman M, El Safadi D, Cian A, Benamrouz S, Nourrisson C, Poirier P, et al. Prevalence and risk factors for intestinal protozoan infections with *Cryptosporidium*, *Giardia*, *Blastocystis* and *Dientamoeba* among schoolchildren in Tripoli, Lebanon. PLoS Negl Trop Dis. 2016;10(3):e0004496. doi: 10.1371/journal.pntd.0004496 26974335 PMC4790957

[48] Aykur M, Calıskan Kurt C, Dirim Erdogan D, Biray Avcı C, Vardar R, Aydemir S, et al. Investigation of *Dientamoeba fragilis* prevalence and evaluation of sociodemographic and clinical features in patients with gastrointestinal symptoms. Acta Parasitol. 2019;64(1):162–170. doi: 10.2478/s11686-018-00017-5 30645736

[49] Karasartova D, Gureser AS, Zorlu M, Turegun-Atasoy B, Taylan-Ozkan A, Dolapci M. et.al Blastocystosis in post-traumatic splenectomized patients. Parasitol Int. 2016;65(6 Pt B):802–805. doi: 10.1016/j.parint.2015.12.004 26697990

[50] Johnson JA, Clark CG. Cryptic genetic diversity in *Dientamoeba fragilis*. J Clin Microbiol. 2000;38(12):4653–4654. doi: 10.1128/JCM.38.12.4653-4654.2000 11101615 PMC87656

[51] Cacciò SM. Molecular epidemiology of *Dientamoeba fragilis*. Acta Trop. 2018;184:73–77. doi: 10.1016/j.actatropica.2017.06.029 28697994

[52] Chan D, Barratt J, Roberts T, Phillips O, Šlapeta J, Ryan U, et al. Detection of *Dientamoeba fragilis* in animal faeces using species specific real time PCR assay. Vet Parasitol. 2016;227:42–47. doi: 10.1016/j.vetpar.2016.07.025 27523936

[53] Cacciò SM, Sannella AR, Manuali E, Tosini F, Sensi M, Crotti D, et al. Pigs as natural hosts of *Dientamoeba fragilis* genotypes found in humans. Emerg Infect Dis. 2012;18(5):838–841. doi: 10.3201/eid1805.111093 22515838 PMC3358053

[54] Stark D, Phillips O, Peckett D, Munro U, Marriott D, Harkness J, et al. Gorillas are a host for *Dientamoeba fragilis*: an update on the life cycle and host distribution. Vet Parasitol. 2008;151(1):21–26. doi: 10.1016/j.vetpar.2007.10.002 18022187

[55] Lankester F, Kiyang JA, Bailey W, Unwin S. Dientamoeba fragilis: initial evidence of pathogenicity in the western lowland gorilla (Gorilla gorilla gorilla). J Zoo Wildl Med. 2010;41(2):350–352. doi: 10.1638/2009-0190.1 20597233

[56] Dogruman-Al F, Simsek Z, Boorom K, Ekici E, Sahin M, Tuncer C, et al. Comparison of methods for detection of *Blastocystis* infection in routinely submitted stool samples, and also in IBS/IBD Patients in Ankara, Turkey. PLoS One. 2010;5(11):e15484. doi: 10.1371/journal.pone.0015484 21124983 PMC2987810

[57] Gough R, Ellis J, Stark D. Comparison and recommendations for use of *Dientamoeba fragilis* real-time PCR assays. J Clin Microbiol. 2019 Apr 26;57(5):e01466–18. doi: 10.1128/JCM.01466-18 30814263 PMC6498029

[58] Roberts T, Barratt J, Harkness J, Ellis J, Stark D. Comparison of microscopy, culture, and conventional polymerase chain reaction for detection of *Blastocystis* sp. in clinical stool samples. Am J Trop Med Hyg. 2011;84(2):308–312. doi: 10.4269/ajtmh.2011.10-0447 21292905 PMC3029188

[59] Stark D, Barratt J, Roberts T, Marriott D, Harkness J, Ellis J. et.al Comparison of microscopy, two xenic culture techniques, conventional and real-time PCR for the detection of *Dientamoeba fragilis* in clinical stool samples. Eur J Clin Microbiol Infect Dis. 2010;29(4):411–416. doi: 10.1007/s10096-010-0876-4 20155433

[60] Stensvold CR, Arendrup MC, Jespersgaard C, Mølbak K, Nielsen HV. Detecting *Blastocystis* using parasitologic and DNA-based methods: a comparative study. Diagn Microbiol Infect Dis. 2007;59(3):303–307. doi: 10.1016/j.diagmicrobio.2007.06.003 17913433

[61] van Lieshout L, Roestenberg M. Clinical consequences of new diagnostic tools for intestinal parasites. Clin Microbiol Infect. 2015;21(6):520–528. doi: 10.1016/j.cmi.2015.03.015 25843505

[62] Stensvold CR, Clark CG. Molecular identification and subtype analysis of *Blastocystis*. Curr Protoc Microbiol. 2016;43:20A.2.1–20A.2.10. doi: 10.1002/cpmc.17 27858971

[63] Jerez Puebla LE, Núñez-Fernández FA, Fraga Nodarse J, Atencio Millán I, Cruz Rodríguez I, Martínez Silva I, et al. Diagnosis of intestinal protozoan infections in patients in Cuba by microscopy and molecular methods: advantages and disadvantages. J Microbiol Methods. 2020;179:106102. doi: 10.1016/j.mimet.2020.106102 33188802

[64] Maloney JG, Molokin A, Santin M. Next generation amplicon sequencing improves detection of *Blastocystis* mixed subtype infections. Infect Genet Evol. 2019;73:119–125. doi: 10.1016/j.meegid.2019.04.013 31026606

[65] Chumpitazi BP, Self MM, Czyzewski DI, Cejka S, Swank PR, Shulman RJ. Bristol Stool Form Scale reliability and agreement decreases when determining Rome III stool form designations. Neurogastroenterol Motil. 2016;28(3):443–448. doi: 10.1111/nmo.12738 26690980 PMC4760857

[66] Garcia LS. Diagnostic medical parasitology. 6th ed. Washington: ASM Press; 2017.

[67] World Health Organization. Basic laboratory methods in medical parasitology: World Health Organization; 1991.

[68] Stensvold CR, Ahmed UN, Andersen LO, Nielsen HV. Development and evaluation of a genus-specific, probe-based, internal-process-controlled real-time PCR assay for sensitive and specific detection of *Blastocystis* spp. J Clin Microbiol. 2012;50(6):1847–1851. doi: 10.1128/JCM.00007-12 22422846 PMC3372105

[69] Stark D, Beebe N, Marriott D, Ellis J, Harkness J. Evaluation of three diagnostic methods, including real-time PCR, for detection of *Dientamoeba fragilis* in stool specimens. J Clin Microbiol. 2006;44(1):232–235. doi: 10.1128/JCM.44.1.232-235.2006 16390978 PMC1351980

[70] Santín M, Gómez-Muñoz MT, Solano-Aguilar G, Fayer R. Development of a new PCR protocol to detect and subtype *Blastocystis* spp. from humans and animals. Parasitol Res. 2011;109(1):205–212. doi: 10.1007/s00436-010-2244-9 21210149

[71] Bushnell B. BBMap: A fast, accurate, splice-aware aligner. Lawrence Berkeley National Lab.(LBNL), Berkeley, CA (United States), 2014.

[72] Rognes T, Flouri T, Nichols B, Quince C, Mahé F. VSEARCH: A versatile open source tool for metagenomics. PeerJ. 2016;4:e2584. doi: 10.7717/peerj.2584 27781170 PMC5075697

[73] Landis JR, Koch GG. The measurement of observer agreement for categorical data. Biometrics. 1977;33(1):159–174. 843571

[74] Stensvold CR, Arendrup MC, Mølbak K, Nielsen HV. The prevalence of *Dientamoeba fragilis* in patients with suspected enteroparasitic disease in a metropolitan area in Denmark. Clin Microbiol Infect. 2007;13(8):839–842. doi: 10.1111/j.1469-0691.2007.01760.x 17610603

[75] Dagci H, Kurt Ö, Demirel M, Mandiracioglu A, Aydemir S, Saz U, Epidemiological and diagnostic features of *Blastocystis* infection in symptomatic patients in Izmir province, Turkey. Iran J Parasitol. 2014;9(4):519–529. 25759733 PMC4345091

[76] Poirier P, Wawrzyniak I, Albert A, El Alaoui H, Delbac F, Livrelli V. et.al Development and evaluation of a real-time PCR assay for detection and quantification of *Blastocystis* parasites in human stool samples: prospective study of patients with hematological malignancies. J Clin Microbiol. 2011;49(3):975–983. doi: 10.1128/JCM.01392-10 21177897 PMC3067686

[77] Moosavi A, Haghighi A, Mojarad EN, Zayeri F, Alebouyeh M, Khazan H, et al. Genetic variability of *Blastocystis* sp. isolated from symptomatic and asymptomatic individuals in Iran. Parasitol Res. 2012;111(6):2311–2315. doi: 10.1007/s00436-012-3085-5 22948205

[78] Tungtrongchitr A, Manatsathit S, Kositchaiwat C, Ongrotchanakun J, Munkong N, Chinabutr P, et al. *Blastocystis hominis* infection in irritable bowel syndrome patients. Southeast Asian J Trop Med Public Health. 2004;35(3):705–710. 15689092

[79] Yoshikawa H, Dogruman-Al F, Turk S, Kustimur S, Balaban N, Sultan N. et.al Evaluation of DNA extraction kits for molecular diagnosis of human *Blastocystis* subtypes from fecal samples. Parasitol Res. 2011;109(4):1045–1050. doi: 10.1007/s00436-011-2342-3 21499752

[80] Nithyamathi K, Chandramathi S, Kumar S. Predominance of *Blastocystis* sp. Infection among School Children in Peninsular Malaysia. PLoS One. 2016;11(2):e0136709. doi: 10.1371/journal.pone.0136709 26914483 PMC4767405

[81] Abdulsalam AM, Ithoi I, Al-Mekhlafi HM, Al-Mekhlafi AM, Ahmed A, Surin J. et.al Subtype distribution of *Blastocystis* isolates in Sebha, Libya. PLoS One. 2013;8(12):e84372. doi: 10.1371/journal.pone.0084372 24376805 PMC3869855

[82] Stensvold CR, Christiansen DB, Olsen KE, Nielsen HV. *Blastocystis* sp. subtype 4 is common in Danish *Blastocystis*-positive patients presenting with acute diarrhea. Am J Trop Med Hyg. 2011;84(6):883–885. doi: 10.4269/ajtmh.2011.11-0005 21633023 PMC3110361

[83] Dogruman-Al F, Yoshikawa H, Kustimur S, Balaban N. PCR-based subtyping of *Blastocystis* isolates from symptomatic and asymptomatic individuals in a major hospital in Ankara, Turkey. Parasitol Res. 2009;106(1):263–268. doi: 10.1007/s00436-009-1658-8 19847459

[84] Yersal O, Malatyali E, Ertabaklar H, Oktay E, Barutca S, Ertug S. et.al *Blastocystis* subtypes in cancer patients: Analysis of possible risk factors and clinical characteristics. Parasitol Int. 2016;65(6 Pt B):792–796. doi: 10.1016/j.parint.2016.02.010 26905740

[85] Coskun A, Malatyali E, Ertabaklar H, Yasar MB, Karaoglu AO, Ertug S. et.al *Blastocystis* in ulcerative colitis patients: Genetic diversity and analysis of laboratory findings. Asian Pac J Trop Med. 2016;9(9):916–919. doi: 10.1016/j.apjtm.2016.07.018 27633310

[86] Dogan N, Aydin M, Tuzemen NU, Dinleyici EC, Oguz I, Dogruman-Al F. et.al Subtype distribution of *Blastocystis* spp. isolated from children in Eskisehir, Turkey. Parasitol Int. 2017;66(1):948–951. doi: 10.1016/j.parint.2016.10.008 27989831

[87] Cakir F, Cicek M, Yildirim IH. Determination the subtypes of *Blastocystis* sp. and evaluate the effect of these subtypes on pathogenicity. Acta Parasitol. 2019;64(1):7–12. doi: 10.2478/s11686-018-00002-y 30649701

[88] Koltas IS, Eroglu F. Subtype analysis of *Blastocystis* isolates using SSU rRNA-DNA sequencing in rural and urban population in southern Turkey. Exp Parasitol. 2016;170:247–251. doi: 10.1016/j.exppara.2016.10.006 27725159

[89] Seyer A, Karasartova D, Ruh E, Güreser AS, Turgal E, Imir T, et al. Epidemiology and prevalence of *Blastocystis* spp. in North Cyprus. Am J Trop Med Hyg. 2017;96(5):1164–1170. doi: 10.4269/ajtmh.16-0706 28167596 PMC5417212

[90] Karaman U, Koloren Z, Ayaz E, Gur U. Epidemiology of *Blastocystis* spp. in primary school students at a central village of Ordu province. Med Sci. 2019;8(1):77–80.

[91] Ostan I, Kilimcioğlu AA, Girginkardeşler N, Ozyurt BC, Limoncu ME, Ok UZ. et.al Health inequities: lower socio-economic conditions and higher incidences of intestinal parasites. BMC Public Health. 2007;7:342. doi: 10.1186/1471-2458-7-342 18042287 PMC2211470

[92] Aksoy U, Akisü C, Bayram-Delibaş S, Ozkoç S, Sahin S, Usluca S. et.al Demographic status and prevalence of intestinal parasitic infections in schoolchildren in Izmir, Turkey. Turk J Pediatr. 2007;49(3):278–282. 17990581

[93] Tan TC, Ong SC, Suresh KG. Genetic variability of *Blastocystis* sp. isolates obtained from cancer and HIV/AIDS patients. Parasitol Res. 2009;105(5):1283–1286. doi: 10.1007/s00436-009-1551-5 19603182

[94] Mohamed AM, Ahmed MA, Ahmed SA, Al-Semany SA, Alghamdi SS, Zaglool DA. et.al Predominance and association risk of *Blastocystis hominis* subtype I in colorectal cancer: a case control study. Infect Agent Cancer. 2017;12:21. doi: 10.1186/s13027-017-0131-z 28413436 PMC5389010

[95] Piubelli C, Soleymanpoor H, Giorli G, Formenti F, Buonfrate D, Bisoffi Z, et al. *Blastocystis* prevalence and subtypes in autochthonous and immigrant patients in a referral centre for parasitic infections in Italy. PLoS One. 2019;14(1):e0210171. doi: 10.1371/journal.pone.0210171 30615638 PMC6322732

[96] Rasti S, Hassanzadeh M, Hooshyar H, Momen-Heravi M, Mousavi SGA, Abdoli A. et.al Intestinal parasitic infections in different groups of immunocompromised patients in Kashan and Qom cities, central Iran. Scand J Gastroenterol. 2017;52(6–7):738–741. doi: 10.1080/00365521.2017.1308547 28362138

[97] Mumcuoğlu I, Coşkun FA, Aksu N, Pürnak T, Güngör C. [Role of *Dientamoeba fragilis* and *Blastocystis* spp. in Irritable Bowel Syndrome]. Turkiye Parazitol Derg. 2013;37(2):73–77 (in Turkish). doi: 10.5152/tpd.2013.19 23955902

[98] Taş Cengiz Z, Akbayram S, Ciçek M, Yilmaz H. [Intestinal parasitoses detected in primary schoolchildren in the Van province]. Turkiye Parazitol Derg. 2009;33(4):289–293 (in Turkish). 20101580

[99] Calik S, Karaman U, Colak C. Prevalence of microsporidium and other intestinal parasites in children from Malatya, Turkey. Indian J Microbiol. 2011;51(3):345–349. doi: 10.1007/s12088-011-0107-4 22754014 PMC3209917

[100] Karaman U, Atambay M, Aycan O, Yologlu S, Daldal N. [Incidence of intestinal parasites in municipal sanitary workers in Malatya]. Turkiye Parazitol Derg. 2006;30(3):181–183 (in Turkish). 17160847

[101] Karaman U, Turan A, Depecik F, Gecit I, Ozer A, Karci E, et al. [Frequency of intestinal parasites among administrators and workers in sanitary and non-sanitary institutions]. Turkiye Parazitol Derg. 2011;35(1):30–33 (in Turkish). doi: 10.5152/tpd.2011.08 21618189

[102] Doğan N, Oz Y, Kocman N, Nursal A. [Comparison of individual differences in the direct microscopic examination in the diagnosis of intestinal parasites]. Turkiye Parazitol Derg. 2012;36(4):211–214 (in Turkish). doi: 10.5152/tpd.2012.51 23339941

[103] Ozçakir O, Güreser S, Ergüven S, Yilmaz YA, Topaloğlu R, Hasçelik G. et.al Characteristics of *Blastocystis hominis* infection in a Turkish university hospital. Turkiye Parazitol Derg. 2007;31(4):277–282. 18224616

[104] Arserim SK, Limoncu ME, Gündüz T, Balcıoğlu IC. Investigation of intestinal parasites in living nursing home. Turkiye Parazitol Derg. 2019;43(2):74–77. doi: 10.4274/tpd.galenos.2019.6321 31204459

[105] Tanyuksel M, Yilmaz H, Ulukanligil M, Araz E, Cicek M, Koru O, et al. Comparison of two methods (microscopy and enzyme-linked immunosorbent assay) for the diagnosis of amebiasis. Exp Parasitol. 2005;110(3):322–326. doi: 10.1016/j.exppara.2005.02.012 15955332

[106] Maçin S, Kaya F, Çağdaş D, Hizarcioglu-Gulsen H, Saltik-Temizel IN, Tezcan I, et al. Detection of parasites in children with chronic diarrhea. Pediatr Int. 2016;58(6):531–533. doi: 10.1111/ped.12959 27322863

[107] Girginkardeşler N, Coşkun S, Cüneyt Balcioğlu I, Ertan P, Ok UZ. *Dientamoeba fragilis*, a neglected cause of diarrhea, successfully treated with secnidazole. Clin Microbiol Infect. 2003;9(2):110–113. doi: 10.1046/j.1469-0691.2003.00504.x 12588330

[108] Gülmez D, Sarıbaş Z, Akyön Y, Ergüven S. [The results of Hacettepe University Faculty of Medicine Parasitology Laboratory in 2003–2012: evaluation of 10 years]. Turkiye Parazitol Derg. 2013;37(2):97–101 (in Turkish). doi: 10.5152/tpd.2013.23 23955906

[109] Sivcan E, Charyyeva A, Ceylan Ş, Yürük M, Erdoğan E, Şahin İ. et.al [*Dientamoeba fragilis* infection in patients with gastrointestinal system complaints]. Mikrobiyol Bul. 2018;52(2):166–179 (in Turkish). doi: 10.5578/mb.66468 29933734

[110] Burgaña A, Abellana R, Yordanov SZ, Kazan R, Pérez Ortiz AM, Ramos CC, et al. Paromomycin is superior to metronidazole in *Dientamoeba fragilis* treatment. Int J Parasitol Drugs Drug Resist. 2019;11:95–100. doi: 10.1016/j.ijpddr.2019.10.005 31759244 PMC6880088

[111] Srichaipon N, Nuchprayoon S, Charuchaibovorn S, Sukkapan P, Sanprasert V. A Simple genotyping method for rapid differentiation of *Blastocystis* subtypes and subtype distribution of *Blastocystis* spp. in Thailand. Pathogens. 2019;8(1):38. doi: 10.3390/pathogens8010038 30901902 PMC6471993

[112] Stensvold CR, Traub RJ, von Samson-Himmelstjerna G, Jespersgaard C, Nielsen HV, Thompson RC. et.al *Blastocystis*: subtyping isolates using pyrosequencing technology. Exp Parasitol. 2007;116(2):111–119. doi: 10.1016/j.exppara.2006.12.002 17266951

[113] Yoshikawa H, Wu Z, Kimata I, Iseki M, Ali IKMD, Hossain MB, et al. Polymerase chain reaction-based genotype classification among human *Blastocystis hominis* populations isolated from different countries. Parasitol Res. 2004;92(1):22–29. doi: 10.1007/s00436-003-0995-2 14598169

[114] Parkar U, Traub RJ, Kumar S, Mungthin M, Vitali S, Leelayoova S, et al. Direct characterization of *Blastocystis* from faeces by PCR and evidence of zoonotic potential. Parasitology. 2007;134(Pt 3):359–367. doi: 10.1017/S0031182006001582 17052374

[115] Scanlan PD, Stensvold CR, Cotter PD. Development and application of a *Blastocystis* subtype-specific PCR assay reveals that mixed-subtype infections are common in a healthy human population. Appl Environ Microbiol. 2015;81(12):4071–4076. doi: 10.1128/AEM.00520-15 25841010 PMC4524157

[116] Yoshikawa H, Iwamasa A. Human *Blastocystis* subtyping with subtype-specific primers developed from unique sequences of the SSU rRNA gene. Parasitol Int. 2016;65(6 Pt B):785–791. doi: 10.1016/j.parint.2016.03.002 26965391

[117] Dogruman-Al F, Dagci H, Yoshikawa H, Kurt O, Demirel M. A possible link between subtype 2 and asymptomatic infections of *Blastocystis hominis*. Parasitol Res. 2008;103(3):685–689. doi: 10.1007/s00436-008-1031-3 18523804

[118] Ozyurt M, Kurt O, Mølbak K, Nielsen HV, Haznedaroglu T, Stensvold CR. et.al Molecular epidemiology of *Blastocystis* infections in Turkey. Parasitol Int. 2008;57(3):300–306. doi: 10.1016/j.parint.2008.01.004 18337161

[119] Forsell J, Granlund M, Samuelsson L, Koskiniemi S, Edebro H, Evengård B. et.al High occurrence of *Blastocystis* sp. subtypes 1–3 and *Giardia intestinalis* assemblage B among patients in Zanzibar, Tanzania. Parasit Vectors. 2016;9(1):370. doi: 10.1186/s13071-016-1637-8 27356981 PMC4928263

[120] Mohamed RT, El-Bali MA, Mohamed AA, Abdel-Fatah MA, El-Malky MA, Mowafy NM, et al. Subtyping of *Blastocystis* sp. isolated from symptomatic and asymptomatic individuals in Makkah, Saudi Arabia. Parasit Vectors. 2017;10(1):174. doi: 10.1186/s13071-017-2114-8 28388938 PMC5383971

[121] Sanpool O, Laymanivong S, Thanchomnang T, Rodpai R, Sadaow L, Phosuk I, et al. Subtype identification of human *Blastocystis* spp. isolated from Lao People’s Democratic Republic. Acta Trop. 2017;168:37–40. doi: 10.1016/j.actatropica.2017.01.006 28088334

[122] Eroglu F, Genc A, Elgun G, Koltas IS. Identification of *Blastocystis hominis* isolates from asymptomatic and symptomatic patients by PCR. Parasitol Res. 2009;105(6):1589–1592. doi: 10.1007/s00436-009-1595-6 19685075

[123] Adiyaman Korkmaz G, Dogruman Al F, Mumcuoglu I. [Investigation of the presence of *Blastocystis* spp. in stool samples with microscopic, culture and molecular methods]. Mikrobiyol Bul. 2015;49(1):85–97 (in Turkish). doi: 10.5578/mb.8439 25706734

[124] Rudzińska M, Kowalewska B, Wąż P, Sikorska K, Szostakowska B. *Blastocystis* subtypes isolated from travelers and non-travelers from the north of Poland—A single center study. Infect Genet Evol. 2019;75:103926. doi: 10.1016/j.meegid.2019.103926 31220611

[125] Clark CG. Extensive genetic diversity in *Blastocystis* hominis. Mol Biochem Parasitol. 1997;87(1):79–83. doi: 10.1016/s0166-6851(97)00046-7 9233675

[126] Rezaei Riabi T, Mirjalali H, Haghighi A, Rostami Nejad M, Pourhoseingholi MA, Poirier P, et al. Genetic diversity analysis of *Blastocystis* subtypes from both symptomatic and asymptomatic subjects using a barcoding region from the 18S rRNA gene. Infect Genet Evol. 2018;61:119–126. doi: 10.1016/j.meegid.2018.03.026 29608961

