# Supplementary material for: Investigation of neglected protists Blastocystis sp. and Dientamoeba fragilis in immunocompetent and immunodeficient diarrheal patients using both conventional and molecular methods
Source: PLoS Negl Trop Dis. 2021 Oct 6;15(10):e0009779. doi: 10.1371/journal.pntd.0009779 (PMC8494357; doi:10.1371/journal.pntd.0009779)
Supplement: S1 Table — (DOCX) [file pntd.0009779.s001.docx]

**S1 Table.** Distribution of immunocompetent and immunodeficient patients according to clinics and protists.

| Immunocompetent patients group | Samples (n) | *Blastocystis* | |  | *Dientamoeba fragilis* | |
| --- | --- | --- | --- | --- | --- | --- |
|  |  | **Positive Samples (n)^1^** | **Prevalence (%)** |  | **Positive Samples (n)^1^** | **Prevalence (%)** |
| Chest Diseases | 1 |  | 0 |  |  |  |
| General Surgery | 2 | 1 | 50,0 |  |  |  |
| Physical Medicine and Rehabilitation | 3 |  | 0 |  |  |  |
| Gynecology and Obstetrics | 3 |  | 0 |  |  |  |
| Endocrinology | 3 | 1 | 33,3 |  |  |  |
| Geriatrics | 4 |  | 0 |  | 1 | 25,0 |
| Internal Medicine | 17 | 6 | 35,3 |  | 2 | 11,8 |
| Infectious Diseases | 24 | 3 | 12,5 |  | 4 | 16,7 |
| Adult Emergency | 28 | 8 | 28,6 |  | 7 | 25,0 |
| Gastroenterology | 108 | 23 | 21,3 |  | 12 | 11,1 |
| *Subtotal* | ***193*** | ***42*** | ***21,8*** |  | ***26*** | ***13,5*** |
| Immunodeficient patients group |  |  |  |  |  |  |
| Rheumatology | 4 |  | 0 |  | 1 | 25,0 |
| Nephrology | 9 | 2 | 22,2 |  |  | 0 |
| Bone Marrow Transplantation | 56 | 4 | 7,1 |  | 3 | 5,4 |
| Medical Oncology | 70 | 6 | 8,6 |  | 7 | 10,0 |
| Hematology | 106 | 19 | 17,9 |  | 15 | 14,2 |
| *Subtotal* | ***245*** | ***31*** | ***12,7*** |  | ***26*** | ***10,6*** |
| Total | **438** | **73** | **16,7** |  | **52** | **11,9** |

^1^ The values of positivity in accordance with the qPCR.
