# Supplementary material for: Investigation of neglected protists Blastocystis sp. and Dientamoeba fragilis in immunocompetent and immunodeficient diarrheal patients using both conventional and molecular methods
Source: PLoS Negl Trop Dis. 2021 Oct 6;15(10):e0009779. doi: 10.1371/journal.pntd.0009779 (PMC8494357; doi:10.1371/journal.pntd.0009779)
Supplement: S2 Table — (DOCX) [file pntd.0009779.s002.docx]

**S2 Table***.* Oligonucleotides which were used for the molecular identification and/or characterization of *Blastocystis* sp., and *Dientamoeba fragilis* in the present study.

| **Target organism** | **Locus** | **Oligonucleotide** | **Sequence (5´–3´)** | **Amplicon size (bp)** | **Reference** |
| --- | --- | --- | --- | --- | --- |
| *Blastocystis* | *SSU* rRNA | Probe | FAM‒TCGTGTAAATCTTACCATTTAGAGGA‒MGB NFQ | 118 | [1] |
|  |  | Blasto_FWD_F5 | GGTCCGGTGAACACTTTGGATTT |  |  |
|  |  | Blasto_R_F2 | CCTACGGAAACCTTGTTACGACTTCA |  |  |
|  | *SSU* rRNA | ILMN_Blast505_532F | TCGTCGGCAGCGTCAGATGTGTATAAGAGACAGGGAGGTAGTGACAATAAATC | 550 | [2] |
|  |  | ILMN_Blast998_1017R | GTCTCGTGGGCTCGGAGATGTGTATAAGAGACAGTGCTTTCGCACTTGTTCATC |  |  |
| *Dientamoeba fragilis* | *SSU* rRNA | Probe | FAM‒CACACCGCCCGTCGCTCCTACCG‒TAMRA | 78 | [3] |
|  |  | DF3 | GTTGAATACGTCCCTGCCCTTT |  |  |
|  |  | DF4 | TGATCCAATGATTTCACCGAGTCA |  |  |

FAM: 6-carboxyfluorescein; MGB: Minor groove binder; NFQ: Nonfluorescent quencher; *SSU* rRNA: small subunit ribosomal RNA; TAMRA: Tetramethylrhodamine.

**References**

1. Stensvold CR, Ahmed UN, Andersen LO, Nielsen HV. Development and evaluation of a genus-specific, probe-based, internal-process-controlled real-time PCR assay for sensitive and specific detection of *Blastocystis* spp. J Clin Microbiol. 2012;50(6):1847-1851.
2. Maloney JG, Molokin A, Santin M. Next generation amplicon sequencing improves detection of *Blastocystis* mixed subtype infections. Infect Genet Evol. 2019;73:119-125.
3. Stark D, Beebe N, Marriott D, Ellis J, Harkness J. Evaluation of three diagnostic methods, including real-time PCR, for detection of *Dientamoeba fragilis* in stool specimens. J Clin Microbiol. 2006;44(1):232-235.
