# Supplementary material for: Investigation of neglected protists Blastocystis sp. and Dientamoeba fragilis in immunocompetent and immunodeficient diarrheal patients using both conventional and molecular methods
Source: PLoS Negl Trop Dis. 2021 Oct 6;15(10):e0009779. doi: 10.1371/journal.pntd.0009779 (PMC8494357; doi:10.1371/journal.pntd.0009779)
Supplement: S3 Table — (DOCX) [file pntd.0009779.s003.docx]

**S3 Table.** Coinfections (n=15) with other enteric parasitic and commensal species detected in the investigated patients.

| Coinfection | *n* | Frequency (%) |
| --- | --- | --- |
| *Blastocystis* sp.*+D.fragilis* | 12 | 80.2 |
| *Blastocystis* sp.*+G. duodenalis* | 1 | 6.6 |
| *Blastocystis* sp.*+Cryptosporidium* spp. | 1 | 6.6 |
| *Blastocystis* sp.*+ Chilomastix mesnili* | 1 | 6.6 |
| Total | 15 | 100 |
