# Supplementary material for: Investigation of neglected protists Blastocystis sp. and Dientamoeba fragilis in immunocompetent and immunodeficient diarrheal patients using both conventional and molecular methods
Source: PLoS Negl Trop Dis. 2021 Oct 6;15(10):e0009779. doi: 10.1371/journal.pntd.0009779 (PMC8494357; doi:10.1371/journal.pntd.0009779)
Supplement: S4 Table — (DOCX) [file pntd.0009779.s004.docx]

**S4 Table.** *Blastocystis* sp. subtypes relative abundance in positive samples identified by next generation sequencing.

| Immunocompetent patients group | | Immunodeficiency patients group | |
| --- | --- | --- | --- |
| Patient ID | **Subtypes (read %)** | **Patient ID** | **Subtypes (read %)** |
| ID/F 016 | ST2(4)*/ST3(96.0) | IY/F 001 | ST3(100) |
| ID/F 018 | ST1(15.5)/ST3(84.5) | IY/F 005 | ST2(100)* |
| ID/F 033 | ST3(100) | IY/F 006 | ST3(100) |
| ID/F 037 | ST3(100) | IY/F 026 | ST3(100) |
| ID/F 038 | ST3(100) | IY/F 034 | ST2(99.7)*/ST3(0.3) |
| ID/F 042 | ST1(99.7)*/ST3(0.3) | IY/F 059 | ST3(100) |
| ID/F 043 | ST2(0.1)/ST3(99.9) | IY/F 074 | ST3(100) |
| ID/F 045 | ST3(100) | IY/F 078 | ST1(99.5)/ST3(0.5) |
| ID/F 047 | ST3(100) | IY/F 082 | ST1(99.8)/ST3(0.2) |
| ID/F 063 | ST3(100) | IY/F 085 | ST3(100) |
| ID/F 067 | ST3(100) | IY/F 100 | ST3(100) |
| ID/F 068 | ST2(91.4)*/ST3(8.6) | IY/F 108 | ST2(100)* |
| ID/F 071 | ST3(100) | IY/F 109 | ST2(100)* |
| ID/F 072 | ST3(100) | IY/F 114 | ST1(100) |
| ID/F 085 | ST3(100) | IY/F 115 | ST3(100) |
| ID/F 087 | ST2(99.8)/ST3(0.2) | IY/F 121 | ST3(100) |
| ID/F 089 | ST3(100) | IY/F 124 | ST2(100) |
| ID/F 091 | ST1(100) | IY/F 133 | ST3(100) |
| ID/F 099 | ST1(99.3)/ST3(0.7) | IY/F 136 | ST3(100) |
| ID/F 100 | ST2(39.2)*/ST3(60.8) | IY/F 156 | ST3(100) |
| ID/F 104 | ST2(100)* | IY/F 170 | ST3(100) |
| ID/F 109 | ST6(100) | IY/F 171 | ST3(100) |
| ID/F 115 | ST3(100) | IY/F 174 | ST6(100) |
| ID/F 125 | ST2(100)* | IY/F 180 | ST2(37.1)*/ST3(62.9) |
| ID/F 129 | ST3(100) | IY/F 185 | ST2(100) |
| ID/F 133 | ST3(100) | IY/F 206 | ST3(100) |
| ID/F 150 | ST2(100) | ID/F 157^1^ | ST2(100)* |
| ID/F 151 | ST4(100) | ID/F 159^1^ | ST3(100) |
| ID/F 158 | ST3(100) |  |  |
| ID/F 164 | ST3(100) |  |  |
| ID/F 165 | ST1(85.8)*/ST3(14.2) |  |  |
| ID/F 169 | ST3(100) |  |  |
| ID/F 170 | ST2(99.7)*/ST3(0.3) |  |  |
| ID/F 173 | ST2(100)* |  |  |
| ID/F 180 | ST2(100) |  |  |
| ID/F 184 | ST1(100)* |  |  |
| ID/F 185 | ST3(100) |  |  |
| ID/F 188 | ST3(100) |  |  |
| ID/F 204 | ST3(100) |  |  |
| ID/F 210 | ST3(100) |  |  |

^*^ Denotes intra-subtype variability

^1^ Randomly encoded in a group of immunocompetent patients
