# Supplementary material for: Investigation of neglected protists Blastocystis sp. and Dientamoeba fragilis in immunocompetent and immunodeficient diarrheal patients using both conventional and molecular methods
Source: PLoS Negl Trop Dis. 2021 Oct 6;15(10):e0009779. doi: 10.1371/journal.pntd.0009779 (PMC8494357; doi:10.1371/journal.pntd.0009779)
Supplement: S5 Table — (DOCX) [file pntd.0009779.s005.docx]

**S5 Table**. *Blastocystis* sp. carriage rates and subtype diversity reported in human samples in Turkey during the period 2000‒2019.

| Province/  Region | Population/sample | No. Samples | Diagnostic method | Carriage rate | Subtyping method | Subtype (ST) (No. isolates) | Reference |
| --- | --- | --- | --- | --- | --- | --- | --- |
| Ankara | Splenectomised patients  Healthy controls | 30  30 | Culture, MC, PCR | 30.0–40.0  13.0 | STS-PCR | ST1 (3), ST3 (3) | [1] |
|  | Patients (with GIS)  Patients (without GIS) | 157  193 | Culture, DFAT, MC, PCR | 19.0  12.0–17.0 | STS -PCR | ST1 (6), ST2 (3), ST3 (12), ST4 (5)  ST6 (1), ST7 (5), Mixed (11) | [2] |
| Aydin | Patients | 100 | MC, PCR | 86.0 | STS -PCR | ST1 (17), ST2 (21), ST3 (50), ST7 (4), ST2/3 (2), ST1/3 (1) | [3] |
|  | Patients (UC) | 150 | Culture, MC, PCR | 4.7–8.0 | BRS - PCR | ST1 (2), ST2 (1), ST3 (8), ST7 (1) | [4] |
|  | Cancer patients | 232 | Culture, MC, PCR | 6.5–10.8 | STS -PCR | ST1 (5), ST2 (4), ST3 (13) | [5] |
|  | Patients | 61 | PCR | 72.1 | STS -PCR | ST1 (9), ST2 (13), ST3 (17), ST1/3 (4), ST1/2 (1) | [6] |
| Diyarbakir | Patients (with GIS, urticaria)  Healthy subjects | 264  81 | MC, PCR | 19.3  14.6 | STS -PCR | ST1 (11), ST2 (11), ST3 (28), ST6 (1), ST1/2 (1), ST1/3 (4), ST2/3 (1), ST1/2/3 (1)  ST1 (1), ST2(1), ST3 (14), ST5 (1), ST1/3 (1) | [7] |
| Eskisehir | Symptomatic children  Asymptomatic children | 84  219 | MC, PCR, qPCR | 21.4  44.3 | STS -PCR | ST1(2), ST3 (7), ST4 (1), ST1/3 (1)  ST1(10), ST2 (4), ST3 (13), ST4 (4), ST1/3 (2), ST1/2 (1), ST2/3(1) | [8] |
| Izmir | Patients (with GIS) | 617 | MC, PCR | 13.5–15.2 | BRS - PCR | ST1 (13), ST2 (11), ST3 (42), ST6 (1), ST7 (1), ST2/3 (2) | [9] |
| Mugla | School children | 468 | MC, PCR | 7.4 | BRS - PCR | ST1 (11), ST2 (9), ST3 (12), ST7(1) | [10] |

BRS: Barcode region sequencing; DFAT: Direct fluorescent antibody test; GIS: Gastrointestinal symptoms; MC: Microscopy; PCR: Polymerase chain reaction; qPCR: Quantitative polymerase chain reaction; STS: Sequence tagged site; UC: ulcerative colitis.

**References**

1. Karasartova D, Gureser AS, Zorlu M, Turegun-Atasoy B, Taylan-Ozkan A, Dolapci M. Blastocystosis in post-traumatic splenectomized patients. Parasitol Int. 2016;65(6 Pt B):802-805.
2. Adıyaman Korkmaz G, Doğruman Al F, Mumcuoğlu İ. [Investigation of the presence of *Blastocystis* spp. in stool samples with microscopic, culture and molecular methods]. Mikrobiyol Bul. 2015;49(1):85-97. Turkish.
3. Malatyalı E, Ertabaklar H, Ertuğ S. [Subtype Distribution of *Blastocystis* spp. with DNA barcoding and evaluation of diagnostic methods]. Mikrobiyol Bul. 2019;53(3):308-318. Turkish.
4. Coskun A, Malatyali E, Ertabaklar H, Yasar MB, Karaoglu AO, Ertug S. *Blastocystis* in ulcerative colitis patients: Genetic diversity and analysis of laboratory findings. Asian Pac J Trop Med. 2016;9(9):916-919.
5. Yersal O, Malatyali E, Ertabaklar H, Oktay E, Barutca S, Ertug S. *Blastocystis* subtypes in cancer patients: Analysis of possible risk factors and clinical characteristics. Parasitol Int. 2016;65(6 Pt B):792-796.
6. Ertuğ S, Malatyalı E, Ertabaklar H, Özlem Çalışkan S, Bozdoğan B. [Subtype distribution of *Blastocystis* isolates and evaluation of clinical symptoms detected in Aydin province, Turkey]. Mikrobiyol Bul. 2015;49(1):98-104. Turkish.
7. Cakir F, Cicek M, Yildirim IH. Determination the subtypes of *Blastocystis* sp. and evaluate the effect of these subtypes on pathogenicity. Acta Parasitol. 2019;64(1):7-12.
8. Dogan N, Aydin M, Tuzemen NU, Dinleyici EC, Oguz I, Dogruman-Al F. Subtype distribution of *Blastocystis* spp. isolated from children in Eskisehir, Turkey. Parasitol Int. 2017;66(1):948-951.
9. Dagci, H., Kurt, Ö., Demirel, M., Mandiracioglu, A., Aydemir, S., Saz, U., et al. Epidemiological and diagnostic features of *Blastocystis* infection in symptomatic patients in izmir province, Turkey. Iran J Parasitol. 2014;9(4):519-529.
10. Sankur F, Ayturan S, Malatyali E, Ertabaklar H, Ertug S. The distribution of *Blastocystis* subtypes among school-aged children in Mugla, Turkey. Iran J Parasitol. 2017;12(4):580-586.
