# Supplementary material for: Investigation of neglected protists Blastocystis sp. and Dientamoeba fragilis in immunocompetent and immunodeficient diarrheal patients using both conventional and molecular methods
Source: PLoS Negl Trop Dis. 2021 Oct 6;15(10):e0009779. doi: 10.1371/journal.pntd.0009779 (PMC8494357; doi:10.1371/journal.pntd.0009779)
Supplement: S6 Table — (DOCX) [file pntd.0009779.s006.docx]

**S6 Table**. *Dientamoeba fragilis* prevalence in human samples in Turkey

| Province/Region | Population/sample | No. Samples | Diagnostic method | Carriage rate % | Reference |
| --- | --- | --- | --- | --- | --- |
| Ankara | IBS patients  Patients with gastroenteritis  Healthy volunteers | 55  80  50 | Culture, TS | 0.0  0.0  0.0 | [1] |
|  | Splenectomised patients  Healthy controls | 30  30 | TS, PCR | 0.0  0.0 | [2] |
|  | Patients with gastrointestinal symptoms  Patients with allergy  Immunocompromised patients  Patients with chronic renal failure  Healthy cases | 475  82  90  23  100 | TS | 3.1  3.6  0.0  0.0  3.0 | [3] |
|  | Primary immunodeficiency patients  Secondary immunodeficiency patients | 24  36 | TS | 8.3  2.7 | [4] |
|  | Patients with gastrointestinal symptoms | 87100 | TS | 0.4 | [5] |
|  | Chronic spontaneous urticaria | 38 | TS | 1.3 | [6] |
|  | Patients with gastrointestinal symptoms | 4303 | TS | 1.4 | [7] |
| Eskisehir | Children with diarrhoea | 225 | TS | 1.3 | [8] |
| Izmir | Patients with diarrhea  Patients without diarrhea | 302  188 | qPCR | 16.3  5.3 | [9] |
| Kayseri | Patients with gastrointestinal symptoms  Healthy controls | 101  20 | qPCR | 11.8  5.0 | [10] |
| Malatya | Children with gastrointestinal symptoms | 1181 | TS | 0.7 | [11] |
|  | Municipal sanitary workers | 241 | TS | 0.8 | [12] |
|  | Administrators and workers in sanitary and non-sanitary institutions | 2264 | TS | 0.9 | [13] |
| Manisa | Nursing home residents | 82 | TS | 2.4 | [14] |
|  | Patients with gastrointestinal symptoms | 400 | TS | 8.8 | [15] |
| Van | Patients with gastrointestinal symptoms | 280 | TS | 3.2 | [16] |
|  | Primary school children | 2975 | TS | 0.4 | [17] |

- GIS: Gastrointestinal symptom; MC: Microscopy; PCR: Polymerase chain reaction; qPCR: Quantitative polymerase chain reaction; TS: Trichrome stain.

**References**

1. Mumcuoğlu I, Coşkun FA, Aksu N, Pürnak T, Güngör C. [Role of *Dientamoeba fragilis* and *Blastocystis* spp. in irritable bowel syndrome]. Turkiye Parazitol Derg. 2013;37(2):73-77. Turkish.
2. Karasartova D, Gureser AS, Zorlu M, Turegun-Atasoy B, Taylan-Ozkan A, Dolapci M. Blastocystosis in post-traumatic splenectomized patients. Parasitol Int. 2016;65(6 Pt B):802-805.
3. Ozçakir O, Güreser S, Ergüven S, Yilmaz YA, Topaloğlu R, Hasçelik G. Characteristics of *Blastocystis* *hominis* infection in a Turkish university hospital. Turkiye Parazitol Derg. 2007;31(4):277-282.
4. Maçin S, Kaya F, Çağdaş D, Hizarcioglu-Gulsen H, Saltik-Temizel N, Tezcan İ, et al. Detection of parasites in children with chronic diarrhea. Pediatr Int. 2016;58(6):531-533.
5. Gülmez D, Sarıbaş Z, Akyön Y, Ergüven S. [The results of Hacettepe University Faculty of Medicine Parasitology Laboratory in 2003-2012: evaluation of 10 years]. Turkiye Parazitol Derg. 2013;37(2):97-101. Turkish.
6. Vezir S, Kaya F, Vezir E, Karaosmanoğlu N, Adiloğlu AK. Evaluation of intestinal parasites in patients with chronic spontaneous urticaria in a territory hospital in Turkey. J Infect Dev Ctries. 2019;13(10):927-932.
7. Sarzhanov F, Köster PC, Dogruman-Al F, Bailo B, Dashti A, Demirel-Kaya F, et al. Detection of enteric parasites and molecular characterization of *Giardia duodenalis* and *Blastocystis* sp. in patients admitted to hospital in Ankara, Turkey. Parasitology. 2021;148(5):550-561.
8. Doğan N, Oz Y, Koçman NU, Nursal AF. [Comparison of individual differences in the direct microscopic examination in the diagnosis of intestinal parasites]. Turkiye Parazitol Derg. 2012;36(4):211-214. Turkish.
9. Aykur M, Calıskan Kurt C, Dirim Erdogan D, Biray Avcı C, Vardar R, Aydemir S, et al. Investigation of *Dientamoeba fragilis* prevalence and evaluation of sociodemographic and clinical features in patients with gastrointestinal symptoms. Acta Parasitol. 2019;64(1):162-170.
10. Sivcan E, Charyyeva A, Ceylan ŞS, Yürük M, Erdoğan E, Şahin İ. [*Dientamoeba fragilis* infection in patients with gastrointestinal system complaints]. Mikrobiyol Bul. 2018;52(2):166-179. Turkish.
11. Calik S, Karaman U, Colak C. Prevalence of microsporidium and other intestinal parasites in children from malatya, Turkey. Indian J Microbiol. 2011;51(3):345-349.
12. Karaman U, Atambay M, Aycan O, Yoloğlu S, Daldal N. [Incidence of intestinal parasites in municipal sanitary workers in Malatya]. Turkiye Parazitol Derg. 2006;30(3):181-183. Turkish.
13. Karaman U, Turan A, Depecik F, Gecit I, Ozer A, Karcı E, et al. [Frequency of intestinal parasites among administrators and workers in sanitary and non-sanitary institutions]. Turkiye Parazitol Derg. 2011;35(1):30-33. Turkish.
14. Arserim SK, Limoncu ME, Gündüz T, Balcıoğlu İC. Investigation of intestinal parasites in living nursing home. Turkiye Parazitol Derg. 2019;43(2):74-77.
15. Girginkardeşler N, Coşkun S, Cüneyt Balcioğlu I, Ertan P, Ok UZ. *Dientamoeba fragilis*, a neglected cause of diarrhea, successfully treated with secnidazole. Clin Microbiol Infect. 2003;9(2):110-113.
16. Tanyuksel M, Yilmaz H, Ulukanligil M, Araz E, Cicek M, Koru O, et al. Comparison of two methods (microscopy and enzyme-linked immunosorbent assay) for the diagnosis of amebiasis. Exp Parasitol. 2005;110(3):322-326.
17. Taş Cengiz Z, Akbayram S, Ciçek M, Yilmaz H. [Intestinal parasitoses detected in primary schoolchildren in the Van province]. Turkiye Parazitol Derg. 2009;33(4):289-293. Turkish.
