# Supplementary material for: Investigation of neglected protists Blastocystis sp. and Dientamoeba fragilis in immunocompetent and immunodeficient diarrheal patients using both conventional and molecular methods
Source: PLoS Negl Trop Dis. 2021 Oct 6;15(10):e0009779. doi: 10.1371/journal.pntd.0009779 (PMC8494357; doi:10.1371/journal.pntd.0009779)
Supplement: S7 Table — (DOCX) [file pntd.0009779.s007.docx]

**S7 Table**. *Blastocystis* sp. subtype diversity based on the molecular data in Turkey.

| Reference | Population/sample | No. Samples | Mono Subtypes | | | | | | | |  | Mixed Subtypes | | | |
| --- | --- | --- | --- | --- | --- | --- | --- | --- | --- | --- | --- | --- | --- | --- | --- |
|  |  |  | **ST1** | **ST2** | **ST3** | **ST4** | **ST5** | **ST6** | **ST7** |  | | **ST1/2** | **ST1/3** | **ST2/3** | **ST1/2/3** |
| [1] | Splenectomised patients  Healthy controls | 30  30 | 3 (50)  - | -  - | 3(50)  - | -  - | -  - | -  - | -  - |  | | -  - | -  - | -  - | -  - |
| [2] | Patients (with GIS)  Patients (without GIS) | 157  193 | 4 (20)  2 (8.7) | 2 (10)  1 (4.3) | 5 (25)  7 (30.5) | 1 (5)  4 (17.4) | -  - | -  1 (4.3) | 4 (20)  1 (4.3) |  | | 4 (20)  7 (30.5) | | | |
| [3] | Patients | 100 | 17 (17.9) | 21 (22.1) | 50 (52.6) | - | - | - | 4 (4.2) |  | | - | 1 (1.1) | 2 (2.1) | - |
| [4] | Patients (UC) | 150 | 2 (16.7) | 1 (8.3) | 8 (66.7) | - | - | - | 1 (8.3) |  | | - | - | - | - |
| [5] | Cancer patients | 232 | 5 (22.7) | 4 (18.2) | 13 (59.1) | - | - | - | - |  | | - | - | - | - |
| [6] | Patients | 61 | 9 (20.5) | 13 (29.5) | 17 (38.6) | - | - | - | - |  | | 1 (2.4) | 4 (9) | - | - |
| [7] | Patients (with GIS, urticaria)  Healthy subjects | 264  81 | 11 (19)  1 (2.55) | 11 (19)  1 (5.55) | 28 (48.3)  14 (77.8) | -  - | -  1 (5.55) | 1 (1.7)  - | -  - |  | | 1 (1.7)  - | 4 (6.9)  1 (5.55) | 1 (1.7)  - | 1 (1.7)  - |
| [8] | Symptomatic children  Asymptomatic children | 84  219 | 2 (18.2)  10 (30.4) | -  4 (12.1) | 7 (63,6)  13 (39.4) | 1 (9.1)  4 (12.1) | -  - | -  - | -  - |  | | -  1 (3) | 1 (9.1)  - | -  1 (3) | -  - |
| [9] | Patients (with GIS) | 617 | 13 (18.6) | 11 (15.8) | 42 (60) | - | - | 1 (1.4) | 1 (1.4) |  | | - | - | 2 (2.8) | - |
| [10] | School children | 468 | 11 (33.3) | 9 (27.3) | 12 (36.4) | - | - | - | 1 (3) |  | | - | - | - | - |
| Present study | Immunodeficient patients  Immunocompetent patients | 245  193 | 1 (3.6)  2 (5) | 6 (21.4)  5 (12.5) | 16 (57.2)  21 (52.5) | -  1 (2.5) | -  - | 1 (3.6)  1 (2.5) | -  - |  | | -  - | 2 (7.1)  4 (10) | 2 (7.1)  6 (15) | -  - |
|  |  |  | Mono Subtypes (n=467) | | | | | | |  | | Mixed Subtypes (n=35) | | | |
| Total |  |  | 93 | 89 | 256 | 11 | 1 | 5 | 12 |  | | 3 | 17 | 14 | 1 |
| % |  |  | 19,9 | 19,1 | 54,8 | 2,4 | 0,2 | 1,1 | 2,6 |  | | 8,6 | 48,6 | 40,0 | 2,9 |

GIS: Gastrointestinal symptoms; UC: ulcerative colitis.

**References**

1. Karasartova D, Gureser AS, Zorlu M, Turegun-Atasoy B, Taylan-Ozkan A, Dolapci M. Blastocystosis in post-traumatic splenectomized patients. Parasitol Int. 2016;65(6 Pt B):802-805.
2. Adıyaman Korkmaz G, Doğruman Al F, Mumcuoğlu İ. [Investigation of the presence of *Blastocystis* spp. in stool samples with microscopic, culture and molecular methods]. Mikrobiyol Bul. 2015;49(1):85-97. Turkish.
3. Malatyalı E, Ertabaklar H, Ertuğ S. [Subtype Distribution of *Blastocystis* spp. with DNA barcoding and evaluation of diagnostic methods]. Mikrobiyol Bul. 2019;53(3):308-318. Turkish.
4. Coskun A, Malatyali E, Ertabaklar H, Yasar MB, Karaoglu AO, Ertug S. *Blastocystis* in ulcerative colitis patients: Genetic diversity and analysis of laboratory findings. Asian Pac J Trop Med. 2016;9(9):916-919.
5. Yersal O, Malatyali E, Ertabaklar H, Oktay E, Barutca S, Ertug S. *Blastocystis* subtypes in cancer patients: Analysis of possible risk factors and clinical characteristics. Parasitol Int. 2016;65(6 Pt B):792-796.
6. Ertuğ S, Malatyalı E, Ertabaklar H, Özlem Çalışkan S, Bozdoğan B. [Subtype distribution of *Blastocystis* isolates and evaluation of clinical symptoms detected in Aydin province, Turkey]. Mikrobiyol Bul. 2015;49(1):98-104. Turkish.
7. Cakir F, Cicek M, Yildirim IH. Determination the subtypes of *Blastocystis* sp. and evaluate the effect of these subtypes on pathogenicity. Acta Parasitol. 2019;64(1):7-12.
8. Dogan N, Aydin M, Tuzemen NU, Dinleyici EC, Oguz I, Dogruman-Al F. Subtype distribution of *Blastocystis* spp. isolated from children in Eskisehir, Turkey. Parasitol Int. 2017;66(1):948-951.
9. Dagci, H., Kurt, Ö., Demirel, M., Mandiracioglu, A., Aydemir, S., Saz, U., et al. Epidemiological and diagnostic features of *Blastocystis* infection in symptomatic patients in izmir province, Turkey. Iran J Parasitol. 2014;9(4):519-529.
10. Sankur F, Ayturan S, Malatyali E, Ertabaklar H, Ertug S. The distribution of *Blastocystis* subtypes among school-aged children in Mugla, Turkey. Iran J Parasitol. 2017;12(4):580-586.
